# Phase prediction and experimental realisation of a new high entropy alloy using machine learning

**DOI:** 10.1038/s41598-023-31461-7

**Published:** 2023-03-23

**Authors:** Swati Singh, Nirmal Kumar Katiyar, Saurav Goel, Shrikrishna N. Joshi

**Affiliations:** 1 Department of Mechanical Engineering, Indian Institute of Technology Guwahati, Guwahati, 781039 India; 2 School of Engineering, London South Bank University, 103 Borough Road, London, SE1 0AA UK; 3 University of Petroleum and Energy Studies, Dehradun, 248007 India

**Keywords:** Mechanical engineering, Computational methods

## Abstract

Nearly ~ 10^8^ types of High entropy alloys (HEAs) can be developed from about 64 elements in the periodic table. A major challenge for materials scientists and metallurgists at this stage is to predict their crystal structure and, therefore, their mechanical properties to reduce experimental efforts, which are energy and time intensive. Through this paper, we show that it is possible to use machine learning (ML) in this arena for phase prediction to develop novel HEAs. We tested five robust algorithms namely, K-nearest neighbours (KNN), support vector machine (SVM), decision tree classifier (DTC), random forest classifier (RFC) and XGBoost (XGB) in their vanilla form (base models) on a large dataset screened specifically from experimental data concerning HEA fabrication using melting and casting manufacturing methods. This was necessary to avoid the discrepancy inherent with comparing HEAs obtained from different synthesis routes as it causes spurious effects while treating an imbalanced data—an erroneous practice we observed in the reported literature. We found that (i) RFC model predictions were more reliable in contrast to other models and (ii) the synthetic data augmentation is not a neat practice in materials science specially to develop HEAs, where it cannot assure phase information reliably. To substantiate our claim, we compared the vanilla RFC (V-RFC) model for original data (1200 datasets) with SMOTE-Tomek links augmented RFC (ST-RFC) model for the new datasets (1200 original + 192 generated = 1392 datasets). We found that although the ST-RFC model showed a higher average test accuracy of 92%, no significant breakthroughs were observed, when testing the number of correct and incorrect predictions using confusion matrix and ROC-AUC scores for individual phases. Based on our RFC model, we report the development of a new HEA (Ni_25_Cu_18.75_Fe_25_Co_25_Al_6.25_) exhibiting an FCC phase proving the robustness of our predictions.

## Introduction

To overcome twenty-first century grand engineering challenges, the investigation of unexplored central region of the ternary phase diagram is indispensable, which occupies the complex multi-component alloys or popularly known high-entropy alloys (HEAs)^1,2^. HEAs have ample compositional space and possess exceptional properties such as excellent mechanical performance at high temperatures, exceptional ductility, high fracture toughness at cryogenic temperatures, bio-compatibility, high conductivity, excellent catalytic and magnetic properties which means that one or more than one HEA can potentially offer a solution for most engineering problems concerning materials^3–5^.

The combination theory suggests a large compositional space (nearly ~ 10^8^ types of HEAs can be developed from about 64 elements in the periodic table) in the central region of the ternary phase diagram^6,7^. However, it is only since 2004 when HEAs were first discovered, and since then, the profound study is compelling to accelerate the pace of discovery of novel HEAs. The search for new HEAs is strenuous, because each element, its weight percentage, various synthesis routes (vacuum arc melting, powder metallurgy, selective laser melting, additive manufacturing and others) and their processing parameters (cooling rate, processing time, temperature, vacuum/gas) can affect the phase in which a high-entropy alloy stabilises^8,9^. The enormity of composition-processing-structure-performance space makes the searches based on the traditional trial-and-error approach extremely difficult and time-consuming.

Traditionally, new high-entropy alloys are recognised using empirical rules, for instance, a series of Ti_x_NbMoTaW (the molar ratio x = 0, 0.25, 0.5, 0.75 and 1) refractory high-entropy alloys were developed to find an alloy that can surpass the elevated temperature properties of Ni-based superalloys for further improvement of the turbine efficiency^8,10^.

Computational tools can fast predict materials, which are enabling rapid advances in materials discovery and beyond through initiatives such as Materials-4.0^11,12^. Hitherto methods such as ab-initio calculations^13,14^, Monte Carlo simulation^15^, and CALPHAD^16,17^ are used in the arena of material prediction for HEAs. Molecular dynamics (MD) & Density functional theory (DFT) methods are two other choices for studying the mechanical behaviour of materials. As with other techniques, these methods have limitations, for instance, DFT requires a large computational power and is limited to few atoms, while MD suffers limitations arising from force-field or inter-atomic potential function to capture the nature of atomic bonding (cocktail effect, lattice distortion, configurational entropy and sluggish diffusion) reported experimentally in HEAs^9^.

Machine learning in particular is on the rise of prominence in the last decade as one can simply make use of the available dataset to discover a general trend^18^. Machine learning (ML) is a subset of artificial intelligence (a technique that enables machines to apply intelligence akin to a human brain), also known as a data-driven approach, which relies on pattern recognition from a given set of data^19,20^. The accuracy of results predicted by ML depends on the extent of data fed to the ML algorithm to train the system^21^. A significant surge in the use of ML in materials research is evidence of the promise this technique offers as explained by the other researchers^22^. Various ML algorithms such as the Artificial neural network, Convolutional neural network, Random Forest, Support vector machine, Decision trees, Gradient boosting, K-nearest neighbour, XGBoost, logistic regression and Naïve Bayes are employed over the past few years in predicting various phases of HEAs.

In all these studies, the known empirical rules for forming solid solution and phase determination have been applied, which include parameters such as atomic size difference (δ), electronegativity difference (∆χ), valence electron concentration (VEC), thermodynamic rule (mixing enthalpy (∆H_mix_) and mixing entropy (∆S_mix_)), and others (Ω-parameter, ϕ‐parameter, and γ-parameter)^23^. These terms describe the associated chemistry underlying the formation of HEAs and provide an insight into phase prediction, which can be mathematically stated as^24^:

1 $${\text{VEC}} = \mathop \sum \limits_{i = 1}^{n} \left( {c_{i} {\text{VEC}}_{i} } \right)$$

2 $$\Delta {\text{S}}_{{{\text{mix}}}} = - R\mathop \sum \limits_{i = 1}^{n} \left( {c_{i} {\text{ln}}c_{i} } \right)$$

3 $$\Delta {\text{H}}_{{{\text{mix}}}} = \mathop \sum \limits_{i = 1, i < j}^{n} \left( {4 H_{ij} c_{i} c_{j} } \right)$$

4 $$\Delta \chi = \sqrt {\mathop \sum \limits_{i = 1}^{n} c_{i} } \left( {x_{i} - \overline{x}} \right)^{2}$$

5 $$\delta = \sqrt {\mathop \sum \limits_{i = 1}^{n} c_{i} } \left( {1 - r_{i} /\overline{r}} \right)^{2}$$

where, *c*_*i*_ is the atomic percentage of the *i*th element, n represents the total number of metallic elements in a high-entropy alloy. *VEC*_*i*_ is the valence electron concentration of the *i*th element, R is the gas constant, *H*_*ij*_ is the mixing enthalpy for the atomic pairs. *x*_*i*_ and *x̄ a*re the Pauling electronegativity and averaged Pauling electronegativity, respectively, *r*_*i*_ is the atomic radius of the *i*th element, and $\overline{r}$ is the average atomic radius. Note that the actual value of δ was multiplied by numerical factor 100 for better clarity. Corroborating these parameters with the historical data have started to gain prominence^25,26^ leading to the emergence of the use of ML to significantly identify, approximate and explain the structure–property relationships in HEAs in a cost-effective manner^12,27^. In the literature concerning phase prediction of HEAs using ML algorithms, no study can be seen that targets one particular synthesis route to extract the data reliably from experimental studies which can help avoid the spurious effect of an alternative synthesis routes on the resulting phase. For example, Bakr et al*.*^28^ used neural network on 775 samples of HEAs synthesized from mixed manufacturing routes (Arc-melting, sintering, SLM, and others) and obtained 93.4% accuracy in predicting the existence of different phases (AM, BCC, FCC, and IM). Their study did not consider the effect of manufacturing method on the resulting phase of HEAs.

Furthermore, in an attempt to balance out the majority and minority class of an imbalanced dataset, various studies have exercised over-sampling and under-sampling methods. This has been done either by supplementing the synthetically generated data to remaining classes for making it equal to the majority class in case of over-sampling method or by subtracting the data from other classes for making it equal to the minority class in case of under-sampling method. Some studies have also utilised generative adversarial network (GAN) for generating synthetic data to avoid the biasness in the dataset. However, whether an alloy may be called as an HEA is controversial. This became the primary basis for our investigation as we believe that synthetic data is not comparable fully with the experimental data and cannot be considered prudent.

In this paper, we formulate the research objectives keeping in mind the current research gaps in the extant literature as below:

- Consolidate the scattered data on HEA synthesis obtained specifically through melting and casting routes such as: induction melting/induction levitation melting/ vacuum induction melting, arc melting/smelting and casting, arc melting + suction casting, electric/vacuum arc melting followed by suction casting techniques and to use this data as a fresh/new dataset for machine learning predictions. As opposed to previously published studies, the dataset used here included ternary, quaternary, quinary, and other alloys with more constituent elements making the algorithm ultra-robust, while targeting a synthesis route (melting + casting) that yields consistent phases during repeat experiments. This helped us avoid the spurious effect when combining the data from different synthesis routes on the resulting phase of a HEA. The dataset we tested was carefully screened from various experimental papers concerning synthesis of 3d-transition metals HEAs, refractory metals HEAs, HEA brasses and bronze, low-density HEAs, and some precious metal HEAs.
- To use a variety of available machine learning algorithms in their vanilla form (base models) such as K-nearest neighbours (V-KNN), support vector machine (V-SVM), decision tree classifier (V-DTC), random forest classifier (V-RFC), and XGBoost (V-XGB) to obtain phase prediction or to classify the phases of new HEAs into solid-solutions (FCC, BCC, FCC + BCC) or mixture of intermetallic phases (MIP) with the view to compare and contrast the robustness of each ML model based on various alternative evaluation metrics in case of imbalanced data, where accuracy percentage can be a misleading indicator.
- Whether synthetic data augmentation is reliable in predicting complex alloys such as HEAs? In testing this fact, we compared the vanilla RFC (V-RFC) model for 1200 original datasets with SMOTE-Tomek links augmented RFC (ST-RFC) model for 192 new datasets and in total 1392 datasets (1200 original + 192 generated = 1392).
- To synthesise HEA based on our ML predictions for proving the need to eliminate computationally/cost intensive approaches such as ThermoCalc, DFT and ab-initio methods in predicting phase of new HEAs.

## Research methodology

Depending on the chemical nature of the constituting elements, HEAs can be classified into five main subfamilies: (i) 3d transition metal high-entropy alloys (3d TM HEAs) having Fe, Ni, Co, Mn, Ti and Cr typically exhibiting face-centered cubic (FCC) solid solutions; (ii) refractory high entropy alloys (RHEAs) constituted by elements of the groups IVB, VB and VIB exhibiting body-centered cubic (BCC) solid solutions; (iii) low-density high-entropy alloys, constituted of light elements like Al, Be, Li, Mg, Ti, Sc, typically presenting hexagonal closed-packed (HCP); and (iv) HEAs constituted by at least four of the lanthanide elements, also exhibiting HCP solid solutions; and (v) other HEAs, exhibiting the formation of multiple chemically disordered solid solutions (with FCC, BCC, or HCP lattice structures), ordered phases as B2 and L21, as well as different intermetallics (such as the σ, μ, C14, C15, and C36 Laves phases, among others)^29^. It suggests that a very scant number of HEAs have been discovered so far. It is timely to unearth the unexplored compositionally concentrated solid solution alloys at a faster pace to develop novel solutions for various engineering problems.

**Figure 1:**
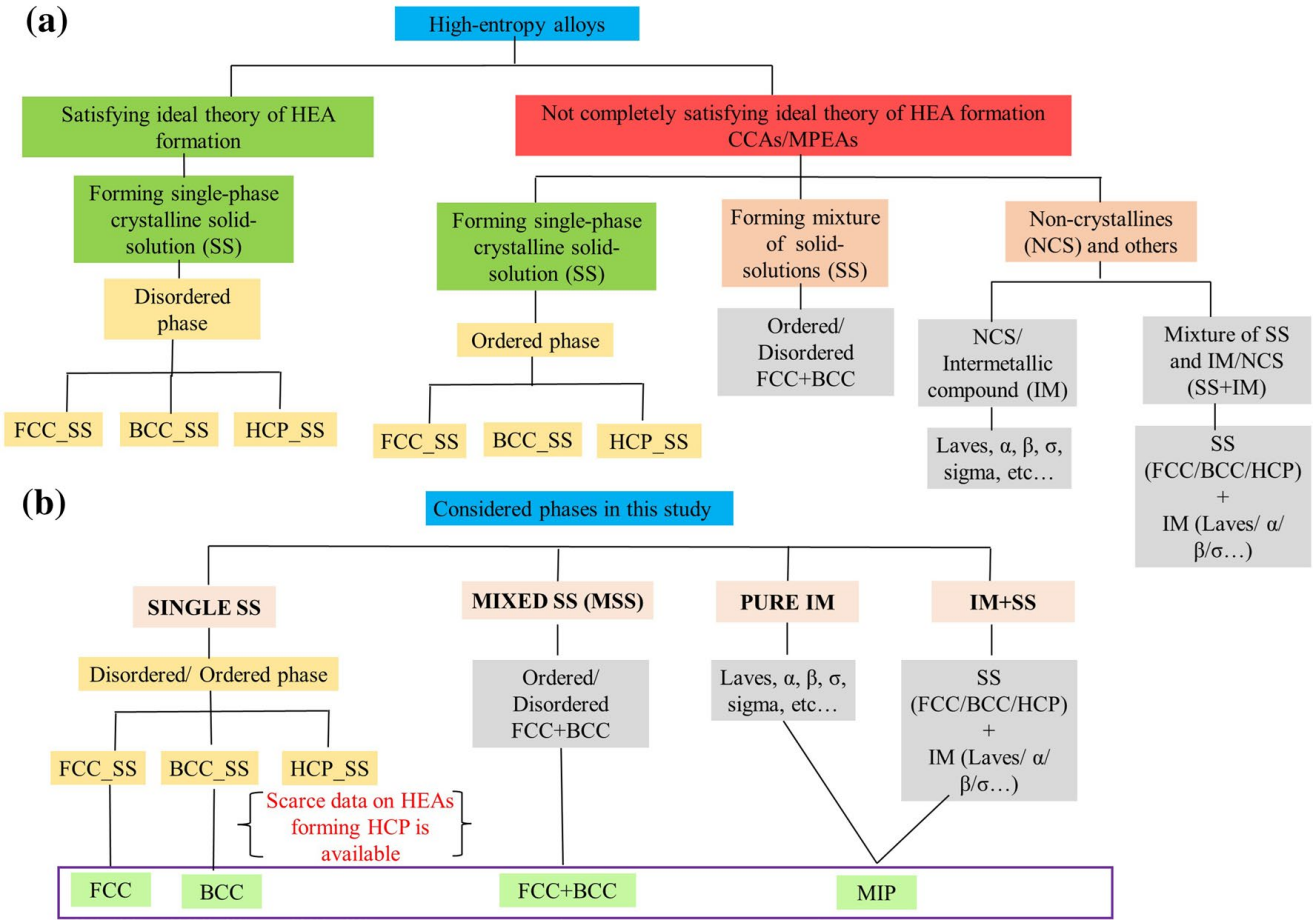
(**a**) Taxonomy of HEAs based on different definitions^30^. (**b**) Phases considered in this work to classify the data based on the existing literature.

HEAs emerged in about 2004 and currently a lot of work is ongoing on their developments. There are however open questions such as what constitutes an HEA. According to Miracle and Senkov^30^, the term HEAs refers to a single-phase solid-solution prepared by controlling the configurational entropy, which limits the objective of exploring the vast compositional space of central region of hyper-dimensional phase diagram. On the other hand, terms such as compositionally complex alloys (CCAs) or multiprincipal element alloys (MPEAs) evokes the vastness of composition space, without concerning the types of phases present or the magnitude of configuration entropy. Figure 1a demonstrates the taxonomy of HEAs based on extant literature, which classifies compositions based on whether they satisfy the ideal theory of HEAs formation or not (called as MPEA/ CCAs).

Various phases in HEA known theoretically to date can be categorised as below:

1. Ordered solid-solution (SS) phase: HEA residing in a singular crystalline phase such as B2 or β-ordered BCC phase
2. Disordered solid-solution (SS) phase: BCC, FCC, HCP
3. Mixed SS (Ordered + Disordered): FCC + BCC, BCC + B2, FCC + B2
4. Pure Intermetallics (IM): α, β, σ, μ, L12, L21, C14, C15, and C36 Laves.
5. IM + SS: BCC + C14 Laves, BCC1 + BCC2 + C15 Laves, BCC + β-ordered BCC, FCC + CoMo2Ni-type IM, FCC + IM and so on.

For the purpose of ML predictions, we clustered these phases together as for instance: (1)+(2) were considered as Single phase solid solution (SS), (3) was considered as Mixed solid solutions (MSS), and (4)+(5) were considered as mixture of intermetallic phases referred as ‘MIP’ as shown in Fig. 1.

Depending on the most-available phases procured from various literature, the current database used in this study contained four phases namely FCC, BCC, FCC + BCC, and MIP (mixture of intermetallic phases), as depicted in Fig. 1b. Due to scarcity of data belonging to HCP solid-solution phase, it was not considered in present study.

**Figure 2:**
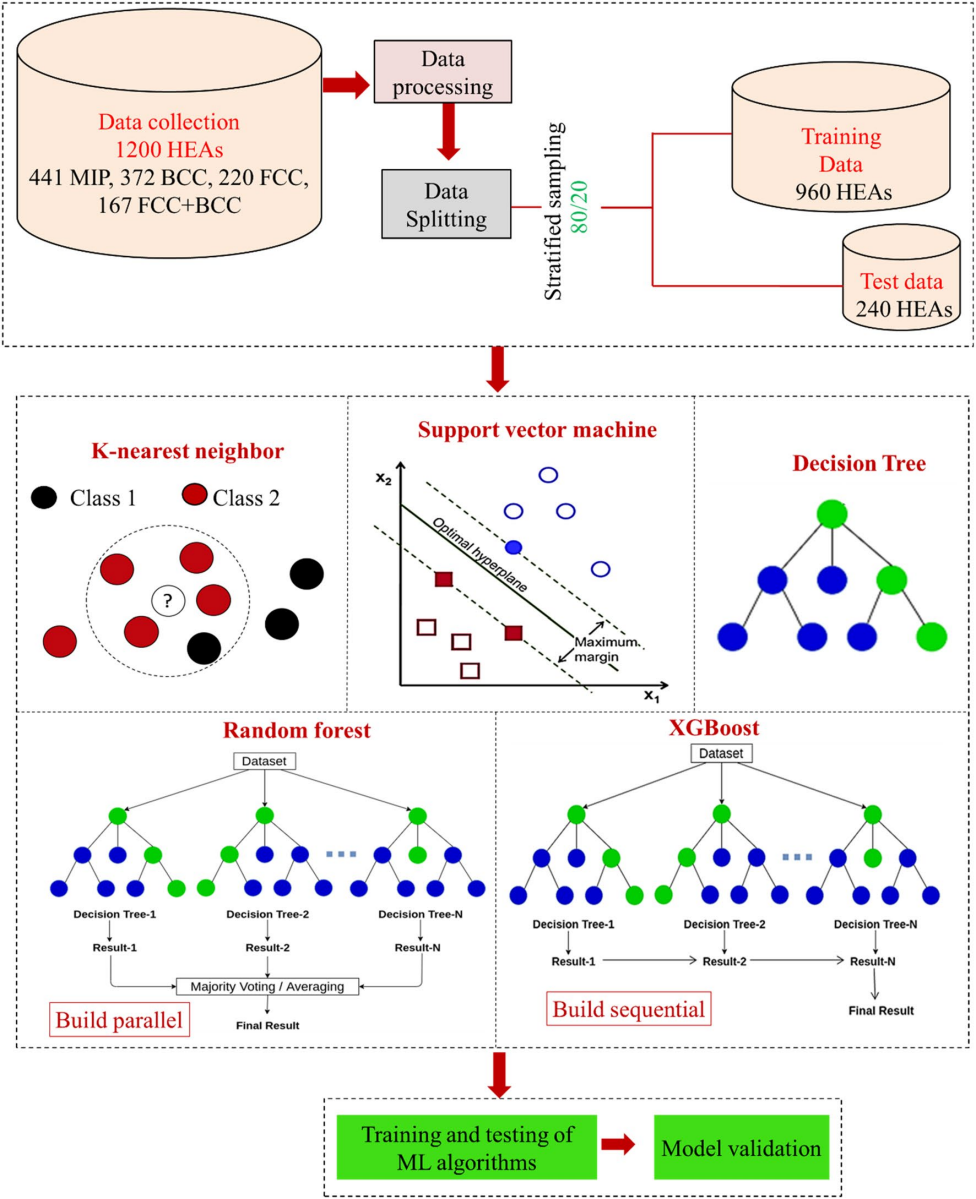
ML framework used in this work for phase prediction of HEAs as solid-solutions (FCC, BCC, FCC + BCC) or mixture of intermetallic phases (MIP).

An open question in the literature is whether we can predict the type of phase (solid-solution, intermetallic, amorphous) for a given composition with known constituent elements, let’s say: Al_x_Co_y_Hf_z_…… alloy, where *x, y, z* is the atomic weight percentage of each element. In this spirit, we demonstrate that ML strategy can be adopted to predict the phase of HEA merely using the reported experimental data by proper training, testing and validation of ML models which has been illustrated through the scheme shown in Fig. 2.

### Data collection

Due to different stoichiometric ratios, distinct synthesis routes or processing conditions adopted by different researchers, the homogeneity in data collection on HEAs cannot be ensured, which makes it a challenging task to consolidate the data for comparison. This study extracted a dataset of 1200 unique compositions of HEAs experimentally synthesised from the melting and casting routes such as induction melting/induction levitation/vacuum induction melting + casting, arc melting/smelting + casting, arc melting + suction casting, or electric/vacuum arc melting followed by suction casting techniques, the corresponding reference to each HEA can be seen from the dataset provided and references^30–32^. The alloys prepared via other synthesis routes (powder metallurgy, selective laser melting, additive manufacturing and others) were not considered to avoid the effect of synthesis route^4^. The current dataset comprises 30 elements (Al, Co, Cr, Fe, Ni, Cu, Mn, Ti, V, Nb, Mo, Zr, Hf, Ta, W, C, Mg, Zn, Si, Re, N, Li, Sn, Be, B, Ag, Pt, Y, Pd, Au) and five physical parameters that are crucial for phase prediction of high-entropy alloys. The range of compositional and physical parameters (minimum, maximum, average and standard deviation values) are tabulated in Table 1. A detailed description of the complete dataset is provided as supplementary information [Table S1 and Fig. S1 in supplementary].

**Table 1:** Range of composition (atomic weight %) and physical parameters used in this study.

Variant	Minimum	Maximum	Average	Deviation
Al	10	100	33.55	24.86
Co	10	99.99	21.58	10.84
Cr	10	100	21.67	11.86
Fe	10	99.99	22.57	10.8
Ni	10	99.99	22	9.64
Cu	10	100	25.76	19.8
Mn	10	89.3	23.8	11.96
Ti	10	100	30.26	21.67
V	10	100	30.23	24.07
Nb	11.11	100	26.2	16
Mo	10	100	29.65	23.98
Zr	10	100	23.9	13.44
Hf	11	100	26.09	21.35
Ta	10	99	23.52	13.1
W	10	90	27.7	19.75
C	10	100	38.5	25.34
Mg	10	71.9	34.88	14.82
Zn	10	52.6	30.79	14.5
Si	10	100	34.63	26.6
Re	10	10	10	NaN
N	19	35	32.33	6.53
Li	10	50	24.75	15.27
Sn	10	99	37.51	25.33
Be	16.7	16.7	16.7	NaN
B	15.4	98.4	50.57	35.32
Ag	16.7	16.7	16.7	NaN
Pt	20	20	20	NaN
Y	11.8	20	16.4	4.01
Pd	20	40	30	14.14
Au	16.7	16.7	16.7	NaN
∆H_mix_ (kJ/mol)	− 166.38	14.82	− 8.61	8.65
∆S_mix_ (J/K mol)	1.609	19.05	12.99	1.905
δ (δ × 100)	0.056	69.93	5.48	3.29
∆χ	0.015	3.92	0.171	0.253
VEC	2.0	10.4	6.74	1.54

Empirical relations observed in high entropy alloys suggest that an HEA (solid-solution phase) formation becomes plausible when δ < 6.6% and 11.6 < ∆H_mix_ < 3.2 kJ/mol. When δ is large (δ > 6.6%) and ∆H_mix_ is noticeably negative (∆H_mix_ = − 12.2 kJ/mol)^24^, it leads to an amorphous phase instead of a crystalline phase. Intermetallic compounds tend to form in the intermediate range in terms of δ and ∆H_mix_, or it overlaps largely with those for solid solutions and amorphous phase. Furthermore, for the identification of crystal structure in various solid solution forming HEAs, the effect of VEC was formulated and the threshold value was found to be as:

- BCC when VEC < 6.87,
- FCC when VEC > 8.0 and
- Mixed phase (BCC + FCC) when VEC is in between 6.87 and 8.0.

A joint plot and swarm plot are shown for better visualisation [Fig. S2 in supplementary]. Zhang et al.^33^ criterion were almost the same for δ (δ < 6.6%) but the range of ∆H_mix_ was slightly different (− 15 < ∆H_mix_ < 5 kJ/mol). Among all physical parameters (atomic size difference (δ), electronegativity difference (∆χ), valence electron concentration (VEC), thermodynamic rule (mixing enthalpy (∆H_mix_) and mixing entropy (∆S_mix_)), and others (Ω-parameter, ϕ‐parameter, and γ-parameter)) proposed for guiding the design of stabilizing phases of HEAs, only five crucial parameters (∆H_mix_, ∆S_mix_, δ, ∆χ, VEC) were considered for this study, as these are widely accepted and easy to compute. Also, the mere requirement of these five parameters which can easily be obtained theoretically, guiding to the development of a new alloy based on our methodology would ensure effortless development of HEAs in future. The ∆H_mix_ for available HEAs in the dataset were calculated using Miedema’s rule^34^, while ∆S_mix_, δ, ∆χ, VEC were calculated by following Guo et al*.*^35^. Other parameters such as geometric parameter (γ) still awaits support from more experimental data. Accordingly, these five most influencing physical parameters being primarily responsible for a crystal structure in HEA were considered in the design of this study.

**Figure 3:**
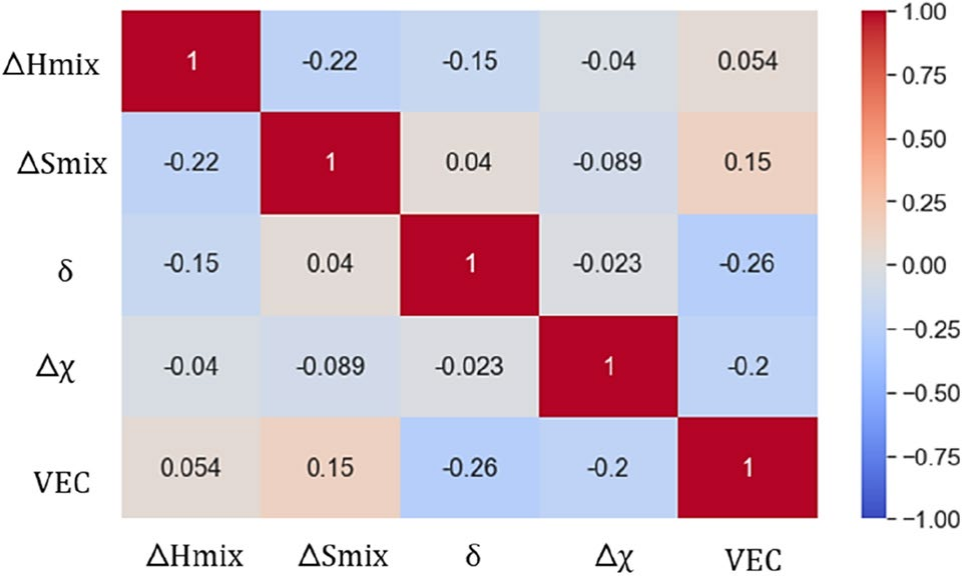
Heatmap showing the Pearson correlation coefficient among five HEA parameters.

As a proof of concept for testing the cruciality of these five parameters, the heatmap shown in Fig. 3 was drawn using the seaborn library of python, which represents the Pearson correlation coefficient of five parameters governing the formation of HEA proposed by various researchers. This heatmap helps to visualize the correlation between features for sanity check of redundant features. Two features that are strongly positively correlated (when two features move in tandem) or negatively correlated (when two features are inversly related) leads to the problem of multicollinearity that significantly reduces the model performance and increases the standard error. Thus, it is suggested to eliminate one of the features that are strongly correlated^36,37^. No such strong positive or negative correlation between any two independent feature was observed; thus, all the five parameters were considered for further study without any elimination.

The complete dataset was labelled in four categories: FCC, BCC, FCC + BCC, and MIP. The alloys with a single-phase ordered/ disordered FCC or multiple FCC such as (FCC1 + FCC2) were considered in ‘FCC’ category. Similarly, alloys with a single-phase ordered/ disordered BCC or multiple BCC such as (BCC1 + BCC2) were considered in ‘BCC’ category and the mixture of FCC and BCC phases was considered in ‘FCC + BCC’ category. Compositions containing pure IM compounds (such as Laves, α, β, sigma etc.) or forming a mixture of SS + IM (such as FCC + IM, FCC + BCC + α, BCC + IM, FCC + α + β, BCC + Laves etc.) were considered in ‘MIP’ category, while the amorphous phase was not included in the analysis.

The 1200 datasets of HEAs used in this work contains 441 compositions of MIP phase, 372 compositions of BCC phase, 220 compositions of FCC phase and 167 compositions of FCC + BCC phase with no duplicated entry of any alloy. Depending on the number of instances belonging to each class, a dataset can typically be recognised as a balanced (when the number of instances available from each class is equal) or an imbalanced dataset (when the number of instances available from each class is different) for a classification problem. In case of an imbalanced dataset, the class with the highest and least number of instances is known as the majority and minority class, respectively.

It must be noted that the present study discusses the phase prediction of HEA as solid solution phases such as (FCC, BCC, or FCC + BCC) or MIP (pure IM or mixed IM + SS) phases for an imbalanced dataset by targeting only those HEAs that were developed via. melting and casting route. The effect that the imbalanced dataset makes on the performance of ML algorithms has been explicitly discussed in section "Results and discussions".

### Data processing

Before feeding the data into the ML algorithms, some statistical processing steps were performed to make the predictions more meaningful^38,39^. The text data (phases) was converted into numeric values (MIP: 0, BCC: 1, FCC: 2, and FCC + BCC: 3), outlier detection was performed to remove the outliers from the dataset; various imputation methods such as simple imputer with different strategies (mean, median, and constant), KNN imputer and MICE imputer was employed to impute the missing values (NaN) in the dataset. Feature scaling was performed on each set of imputed data to normalise the data into a finite range, using robust scalar imported from scikit-learn library. The robust scaling formula can be expressed as^38^:

6 $${X}_{\text{robust }}=\frac{{X}-{X}_{median }}{{X}_{75 }- {X}_{25}}$$

where *X* is an input variable, ${X}_{median}$ is the median of *X*, ${X}_{75}$ is the 75th quantile and ${X}_{25}$ is the 25th quantile of X. The difference between 75th quantile and 25th quantile is also known as interquartile range (IQR).

### Brief description of the machine learning algorithms

#### KNN algorithm

The KNN algorithm searches for the nearest neighbours by measuring the distance between the two points^40,41^ and is expressed as:

7 $$d\left(q,{x}_{i}\right)=\sum_{f\in F} {w}_{f}\delta \left({q}_{f},{x}_{{i}_{f}}\right)$$

For classifying an unknown input variable (*q*) one needs to know the existing input variable (*x*_*i*_) in *F* and the weight factor (w_f_) for each feature. Based on this distance, the *k* nearest neighbours is selected, and the class of *q* is determined from the voting of the nearest neighbours as below:

8 $${\text{Vote}}\left({y}_{i}\right)=\sum_{c=1}^{k} \frac{1}{d\left(q,{x}_{c}\right)\rho }1\left({y}_{i},{y}_{c}\right)$$

This returns 1 if the class labels matches and 0 if does not match. The vote assigned to class *y*_*i*_ by neighbour *x*_*c*_ is the inverse of their distance, i.e., 1(*y*_*i*_*, y*_*c*_).

#### SVM algorithm

SVM classifier searches for the hyper plane that best separates different classes by maximising the margin (the distance between the nearest data points from different class sets) to avoid the local minima and to achieve the best separation of different classes^42,43^. The decision function is as below:

9 $$f\left(x\right)=w.x+b$$

10 $$\underset{\mathbf{w},\xi }{min} \left\{\frac{1}{2}\parallel \mathbf{w}{\parallel }^{2}+C\sum_{i=1}^{N} {\xi }_{i}\right\}$$

11 $${\text{Subject to}}:{ }y_{i} \left( {{\mathbf{w}} \cdot {\mathbf{x}}_{{\mathbf{i}}} } \right) \ge 1 - \xi_{i} ,\xi_{i} \ge 0$$

#### DTC algorithm

A decision tree classifier splits the dataset into root node, sub-node and leaf-node by calculating the information gain, i.e., change in entropy after dividing a dataset based on attributes, which helps to determine the order of features in various nodes of a decision tree (quality of splitting)^44,45^. Information gain is calculated as below:

12 $$H\left( {Y|X} \right) = H\left( {X,Y} \right) - H\left( X \right)$$

where $H(Y\mid X)$ is the conditional entropy, $H\left(X\right)$ is the entropy of random variable X, and $H\left(X,Y\right)$ is the joint entropy, calculated as follows:

13 $$H(X,Y)=-\sum_{i,j} p\left({x}_{i},{y}_{j}\right){\text{log}}_{2}p\left({x}_{i},{y}_{j}\right)$$

#### RFC algorithm

In a random forest classifier, ensembles of various decision trees (base learners) are considered such as ${h}_{1}(x),{h}_{2}(x),\dots ,{h}_{J}(x)$. It takes majority of votes to calculate *f(x)* such that the loss function is minimised^46,47^. Loss function is expressed as below:

14 $$L(Y,f(x))=I(Y\ne f(x))=\left\{\begin{array}{c}0, \, {\text{if}} \, Y=f(x)\\ 1, \, {\text{otherwise}}\end{array}\right.$$

15 $${\text{Voting is based on }}f(x)={\text{arg}}max\sum_{j=1}^{J} I\left(y={h}_{j}(x)\right)$$

#### XGBoost algorithm

XGBoost combines a set of weak classifiers to create a strong classifier^42,48^. The objective function is expressed as:

16 $${\text{obj}}(\theta )=\sum_{i}^{n} l\left({\widehat{y}}_{i},{y}_{i}\right)+\sum_{k}^{K}\Omega \left({f}_{k}\right)$$

The term $l\left({\widehat{y}}_{i},{y}_{i}\right)$ represents the loss function, which measures the difference between predicted output and the actual output, where ${y}_{i}$ is the actual output, and ${\widehat{y}}_{i}$ is the predicted output given by ${\widehat{y}}_{i}=\sum_{k}^{K} {f}_{k}\left({x}_{i}\right),{f}_{k}\in F.$ ${x}_{i}$ is the input variable and $\Omega \left({f}_{k}\right)$ is regularisation term that helps to avoid overfitting by penalising the complexity of the model. XGBoost is trained additively, where one tree is optimised and added each at a time. Supplementary provides the description and proper visualisation of these algorithms [Table S2 in supplementary].

## Results and discussions

For an imbalanced dataset problem such as the one tested in this work, careful treatment is essential or else the predictions can be out of order. Accuracy is well-accepted measurement for evaluating the performance of a classification problem. However, for an imbalanced dataset, the use of accuracy as an effective indicator has been questioned recently by various authors^49–53^. Therefore, alternative evaluation metrics for assessing the effectiveness of ML models for imbalanced dataset were explored, as accuracy alone is not trustworthy. Various other evaluation metrics such as ROC-AUC score, Precision, Recall, and F1-score available in scikit-learn version 1.1.1 module^54^ in python version 3.9.12 are robust measures for imbalanced dataset classification^55^.

The receiver operating characteristic (ROC) curve is a probability curve typically plotted for binary classification tasks at different classification threshold values^54,56^.

This paper studies the phase prediction of HEAs as solid-solution phases such as FCC, BCC, and FCC + BCC, or MIP (which can be either pure IM or mixed IM + SS phase, as described in Fig. 1a), by targeting the multiclass classification of HEAs into four phases namely FCC, BCC, FCC + BCC and MIP using the real-world imbalanced dataset of HEAs.

The ROC curve can be extended to multiclass classification with ‘one-vs-one’ and ‘one-vs-rest’ strategies^54,57^. Here, ‘one-vs-rest’ strategy was employed, to compute the AUC score (by calculating the area under the ROC curve) for each class against the rest of the class and by subsequently taking its average. ROC_AUC score provides a summary of classifier’s performance by measuring the area under the ROC curve, which is more likely to be true representative of model’s performance. ROC_AUC score varies in between 0 to 1, where 1 denotes the perfect classifier, while 0 denotes a perfectly incorrect classifier.

Precision evaluates the fraction of predicted positives that were actually true positives (TP), Recall determines the ability of a model to predict the true positives (TP), and F1-score calculate the harmonic mean of Precision and Recall^58,59^. The detailed description of Precision, Recall and F1-score are given in supplementary [Table S3 in supplementary].

**Figure 4:**
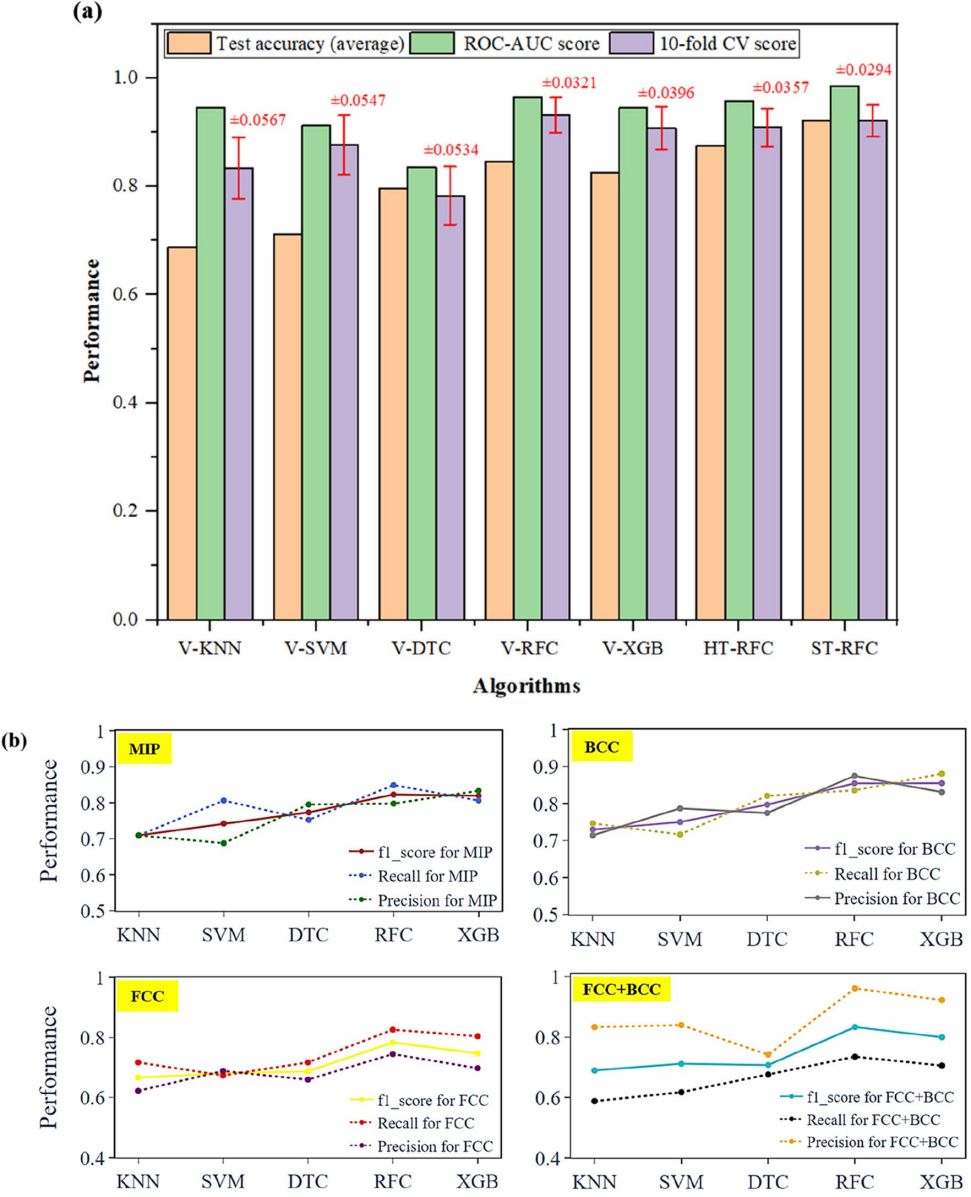
(**a**) Performance comparison of V-KNN, V-SVM, V-DTC, V-RFC, V-XGB, HT-RFC, and ST-RFC models using average test accuracy (multiply by 100 for % value), ROC_AUC score, tenfold cross-validation score and its standard deviation (values shown in red color), (**b**) F1-score, Recall and Precision for four distinct phases of HEAs for five vanilla models.

The effectiveness of five vanilla (base) models (V-KNN, V-SVM, V-DTC, V-RFC, and V-XGB) was tabulated and compared using all the above-mentioned evaluation metrics, for five different imputers (Simple imputer (SI) with strategy mean, median and constant; KNN imputer and MICE imputer), tabulated in supplementary [Table S4]. No significant difference in model’s performance for these imputers were observed. Vanilla-RFC (V-RFC) performed best compared to other algorithms, with an average test accuracy of 84%, ROC-AUC score of 0.9649, tenfold cross-validation mean score of 0.9315 which is shown in Fig. 4a.

Furthermore, F1-score, Recall and Precision were also evaluated for all five vanilla models, where V-RFC obtained higher precision, recall and f1-score in contrast to other models (see Fig. 4b). Note that for each model, five iterations were performed and their average was considered. A bar-chart comparing the performance of all ML models tested in this work compares the three outcomes (Fig. 4a), namely, Accuracy (peach-coloured bars), ROC-AUC score (light green bars) and tenfold cross-validation score (light purple bars). We further explored hyper-parameter tuning of RFC model (HT-RFC) and noticed an increment of approximately 3% in average test accuracy (87.49%).

It is not surprising that many studies have reported higher accuracy from their ML predictions but the fact that these accuracies have come through the aid of synthetic data by mixing with the experimental data cast doubts on the reliability of these models. For instance, Risal et al*.*^60^ obtained 92.31% accuracy with higher ROC-AUC, precision, recall and f1-scores but they used over-sampling/under-sampling method to balance out the majority and minority class data by augmenting it with synthetic data while acknowledging that the “ML algorithms usually do not perform well for imbalanced dataset”.

In our considerations, augmenting or polluting the real-world data with synthetically generated data is not reliable for two reasons: first accuracy alone is not the most robust measure for assessing the performance of the ML model for an imbalanced data and second, the controversy on calling an alloy as HEA still exists, thus it cannot be assured that the generated samples are truly a high-entropy alloy.

Still, as per current vogue, we tried to resample our data using SMOTE-Tomek links method for V-RFC model (outperformed among other vanilla algorithms), which is quite different from other existing over-sampling and under-sampling methods. It generates synthetic data for minority class using SMOTE and removes the data from majority class that is closest to minority class using Tomek links^61^. An average of 92% accuracy was observed for augmented data (1200 + 192 = 1392) using SMOTE-Tomek links (ST-RFC), by generating 192 synthetic data.

**Figure 5:**
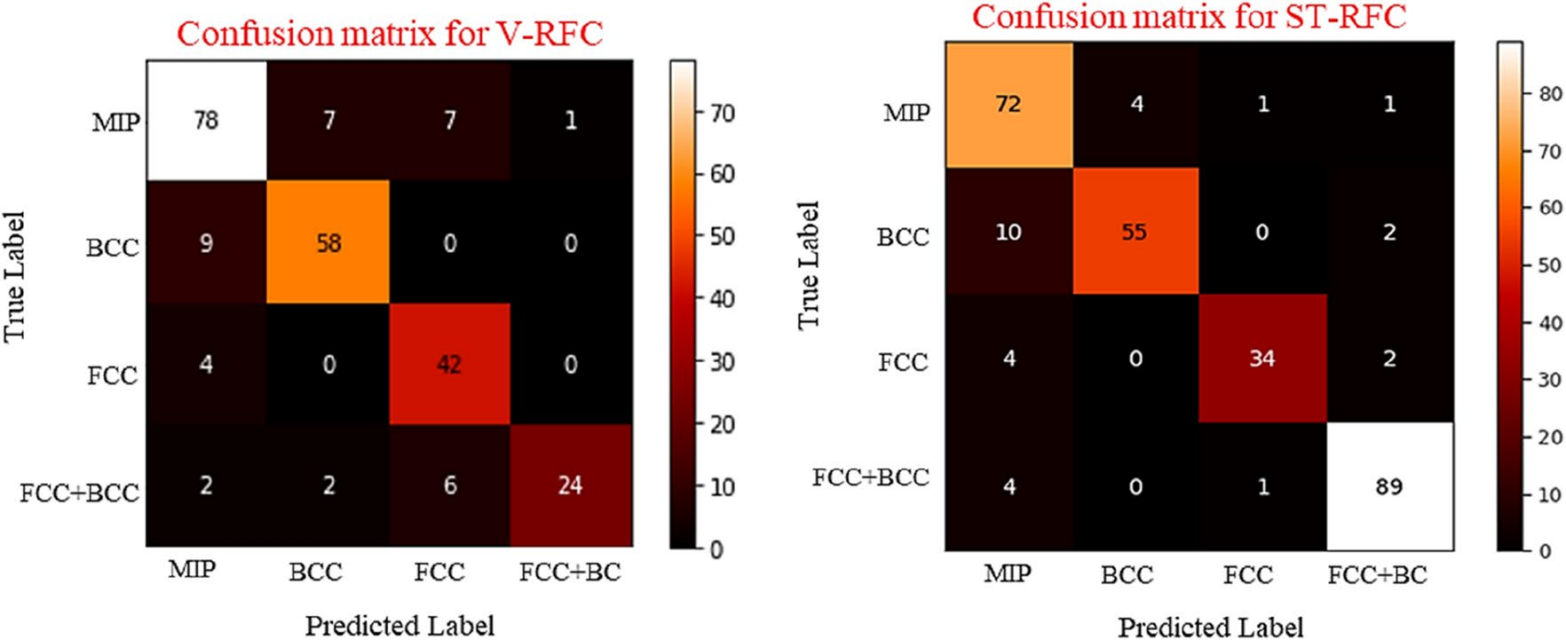
Confusion matrix comparing the performance of V-RFC, and ST-RFC for each distinct phase.

We further evaluated the performance of V-RFC and ST-RFC with a confusion matrix to analyse it’s prediction quality for each phase. A confusion matrix is an easy way to visualise classifier’s performance, where *n* × *n* matrix is created (n is the number of classes) to provide better insights into the correctly and incorrectly classified instances. Two confusion matrices of 4 × 4 were created on test data of 240 HEA samples (93 MIP, 67 BCC, 46 FCC, and 34 FCC + BCC) for V-RFC model from the original dataset (1200), and 279 HEA samples (78 MIP, 67 BCC, 40 FCC, and 94 FCC + BCC) for ST-RFC model from augmented dataset (1200 + 192 = 1392 samples) to investigate the performance of the RFC model in predicting phases of HEAs, as shown in Fig. 5.

**Figure 6:**
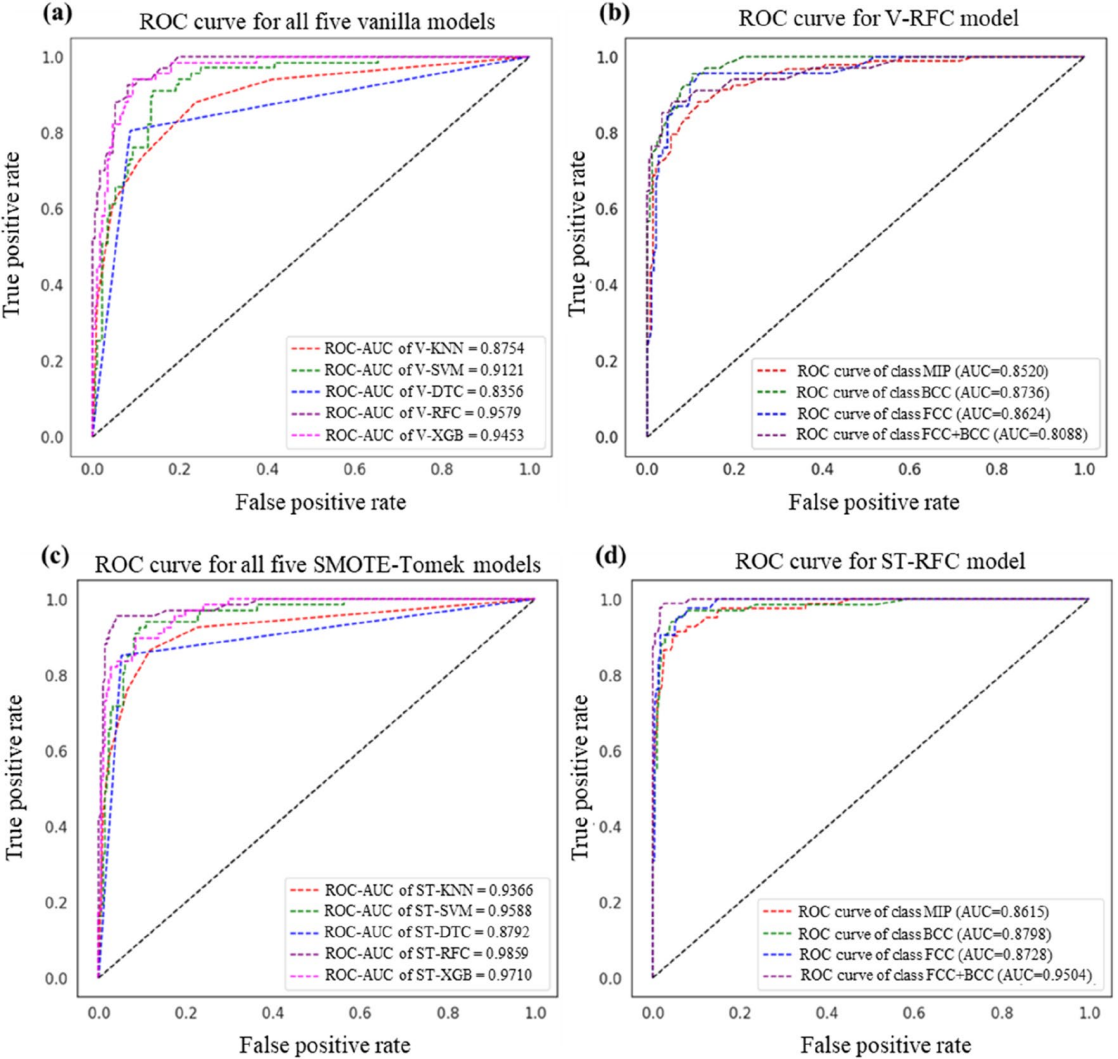
ROC-AUC scores for (**a**) five vanilla (base) models, (**b**) AUC score of each phase for the best vanilla model i.e., V-RFC model, (**c**) for all five SMOTE-Tomek links augmented model, (**d**) AUC score of each phase for the best SMOTE-Tomek links augmented model i.e., ST-RFC model.

It can be noticed that the number of samples in the minority class increases by maintaining the stratified ratio between the classes for the ST-RFC model. Although the number of incorrect predictions becomes less in the case of ST-RFC model in contrast to the V-RFC model, it is still ineffective considering the uncertainty associated with using synthetic data which cannot guarantee a high-entropy alloy. Furthermore, the ROC curve and their AUC score for all five vanilla models trained on original data, and SMOTE-Tomek links models trained on augmented data were plotted as shown in Fig. 6a,c. The ROC-AUC score of all ST-models were higher than the vanilla models. We selected the best models i.e., V-RFC and ST-RFC models and evaluated AUC score for each phase (MIP, BCC, FCC, and FCC + BCC) for the test data of original dataset (test data = 240 HEAs) and augmeneted dataset (test data = 279 HEAs) which are depicted in Fig. 6b,d. It can be seen that although the ROC-AUC of ST-RFC model was approximately 3% higher than the vanilla model (V-RFC), still both models provided approximately similar AUC score for each phase except for the FCC + BCC phase. The reason of higher AUC score for FCC + BCC phase for ST-RFC model is the increased number of instances of minority class i.e., FCC + BCC (34 instances), which has now become the majority class (94 instances) by augmenting the data in case of ST-RFC model. Therefore, we reinforced our point by comparing the confusion matrices and ROC-AUC score for original and augmented dataset. As these matrices provided better insights and are considered as true indicator of a classification model, we claim that augmenting data to increase model’s accuracy is not a reliable practice. Therefore, this study is more pertinent considering the aforementioned issues.

## Model validation

### Validation based on the literature

The predictive capability of all five classifiers was further tested for alloys that were not considered for training or testing the dataset for sense check. Various phases of five alloys (2 refractory HEAs^10^, one 3d-transition metal HEA^62^ and 2 precious metal HEAs^63^) that are recently reported were taken as examples from experimental studies (literature) that are shown in Table 2. The physical parameters corresponding to these HEAs were calculated using the chemical formulae mentioned previously in earlier sections. The phase of HEA highlighted in bold fonts indicated wrong predictions (predictions does not match with experimentally characterised phase), the italicized phases show exceptional case (where the certainty of matching ML predictions and the actual phase is limited), and the remaining phases (nonbold and nonitalic) show the correct prediction revealing that the ML models corroborate with the experimentally reported phases.

**Table 2:** Validation of all vanilla (base) model’s performance for unseen compositions (not used in training or test datasets).

Alloys	Physical parameters					Predicted phases			Actual phases		
	∆H_mix_ (kJ/mol)	∆S_mix_ (J/K.mol)	VEC	∆χ	δ	KNN	SVM	DTC	RFC	XGB	
Refractory HEAs^10^											
Ti_0.5_NbMoTaW	− 3.06	13.15	5.33	0.361	2.6	BCC	**FCC**	BCC	BCC	BCC	BCC
TiNbMoTaW	− 3.04	13.38	5.2	0.356	2.75	BCC	BCC	BCC	BCC	BCC	BCC
3d transition metal HEAs^62^											
Al_0.5_CrCuNiV	− 6.01	13.15	5.43	0.133	4.39	**BCC**	**BCC**	**BCC**	*MIP*	**BCC**	FCC + 2BCC + ordered B2 phase
Precious metal HEAs^63^											
PdPtRhIrCuNi	− 2.56	14.89	9.82	0.161	3.71	FCC	**BCC**	**FCC + BCC**	FCC	FCC	FCC
AuPdAgPtCuNi	− 2.22	14.89	10.49	0.236	5.39	**FCC + BCC**	FCC	**FCC + BCC**	FCC	FCC	FCC

Phases shown in bold font indicate the wrong prediction, italic font shows an exceptional case, and the remaining (nonbold and nonitalic) indicate the correct prediction.

It can further be noted that the RFC classifier predicted the phases correctly in most cases. In case of Al_0.5_CrCuNiV 3d-transition metal HEA^62^, RFC model predicted MIP phase (in italic font), while the actual phase contains 1FCC + 2BCC + ordered B2 phase, which is a complex multiphase alloy. The ML models assumed that MIP can be either pure intermetallic compound (IM) or mixtures of intermetallic and solid solutions (IM + SS) which was discussed in section "Research methodology". Assuming that it can be inferred that the RFC model’s prediction is correct for all new compositions taken from different experimental studies, that were not the part of either training or test dataset. However, it is limited in inferring the number and types of phases present in complex multiphase HEAs. A list of such complex multiphase HEAs (that were not the part of training or test set) is tabulated in Table S5 in supplementary as an additional information.

**Figure 7:**
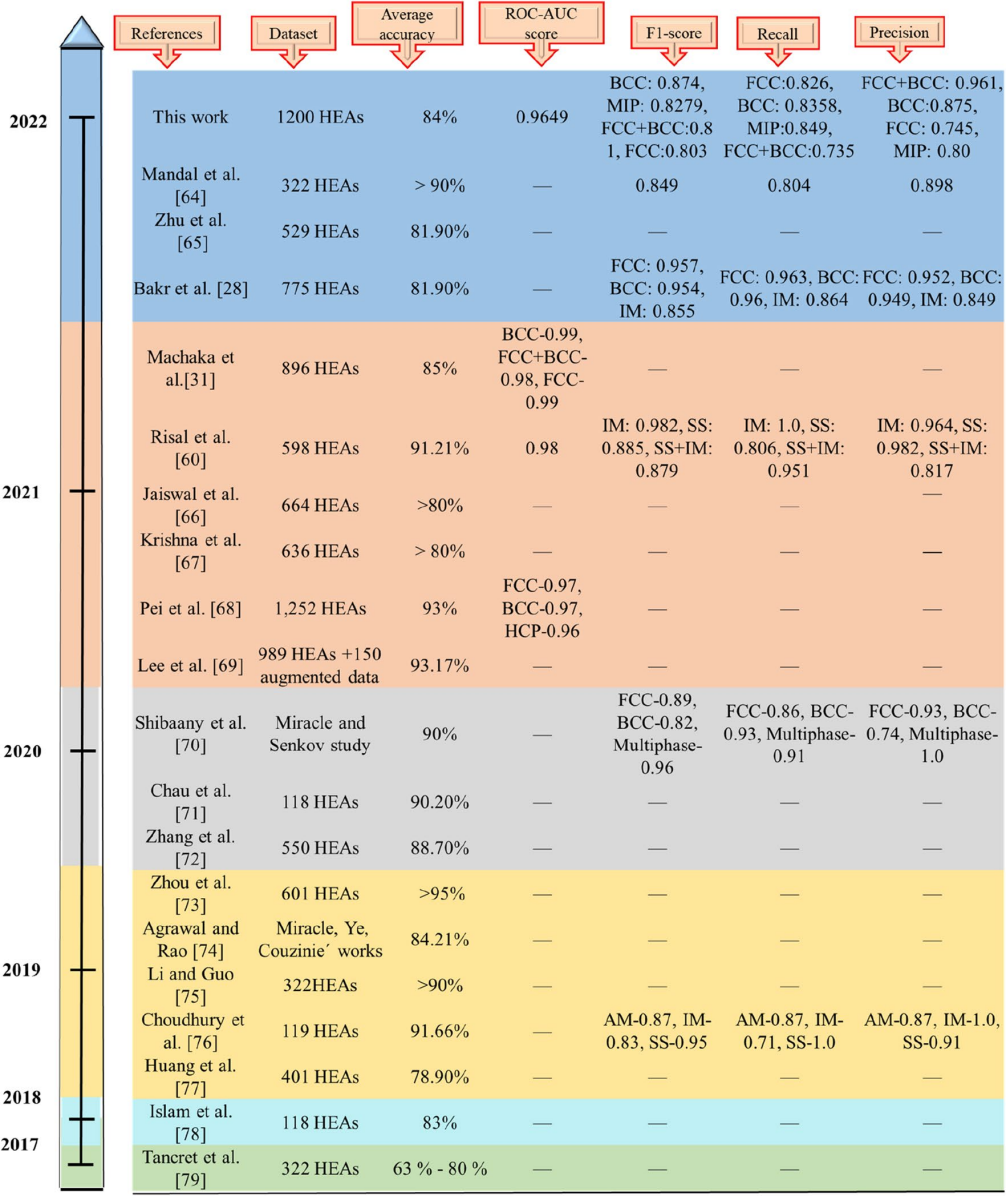
Performance comparison with existing literature^28,31,60,64–79^.

To strengthen the support to our claim further, we critically assessed the recently published literature based on various evaluation metrics, shown in Fig. 7. Scant literature was found to focus on alternative evaluation metrics such as ROC-AUC, precision, recall and F1-score. The proposed RFC model in the present work, revealing the ROC-AUC score, their tenfold cross-validation score, confusion matrix, F1-score, Recall, and Precision, showed satisfactory performance in predicting phases of HEAs as solid-solution phases (BCC, FCC, FCC + BCC) or MIP (denoting either pure intermetallic compounds (IM : such as α, β, σ , L12, C14, C15, C36 Laves, and others) or mixture IM + SS phases such as FCC + IM, FCC + BCC + α, BCC + IM, FCC + α + β, BCC + Laves, BCC1 + BCC2 + C15 Laves etc.)) for large imbalanced dataset of those HEAs that were synthesized via. melting and casting route only, without augmenting/polluting experimental data with generated ones.

### Synthesis and characterisation of a new HEA (Ni_25_Cu_18.75_Fe_25_Co_25_Al_6.25_)

In accord with these learnings, a new high entropy alloy was synthesised based on the predictions obtained from the RFC algorithm. This alloy consists of Nickel, Copper, Iron, Cobalt and Aluminium with a composition of Ni_25_Cu_18.75_Fe_25_Co_25_Al_6.25_.

To begin the experimental synthesis, the metal buttons were procured. Various metal buttons of Ni, Cu, Fe, Co and Al elements (purities > 99.99%) were purchased from Thermofisher Scientific®. All elemental metals were melted together by vacuum arc melting under inert gas (high purity Ar) environment. The ingot formed in the process was melted and solidified multiple times to ensure chemical homogeneity, and then the HEA button was vacuum sealed in a quartz tube, homogenised at 1000 °C for 10 h, and then quenched into water for stabilising high-temperature phase. The detailed description of the newly synthesised high-entropy alloy is specified in Table 3.

**Table 3:** Detailed description of newly synthesized high-entropy alloy.

Novel high-entropy alloy (Ni_25_Cu_18.75_Fe_25_Co_25_Al_6.25_)				
Ni	Cu	Fe	Co	Al
Chemical composition (wt %)				
0.25	0.1875	0.25	0.25	0.0625

Novel high-entropy alloy (Ni_25_Cu_18.75_Fe_25_Co_25_Al_6.25_)				
∆Hmix (kJ/mol)	∆Smix (J/K mol)	VEC	∆χ	δ
Calculated physical parameters				
0.2656	12.689	9	0.07178	3.5973

**Figure 8:**
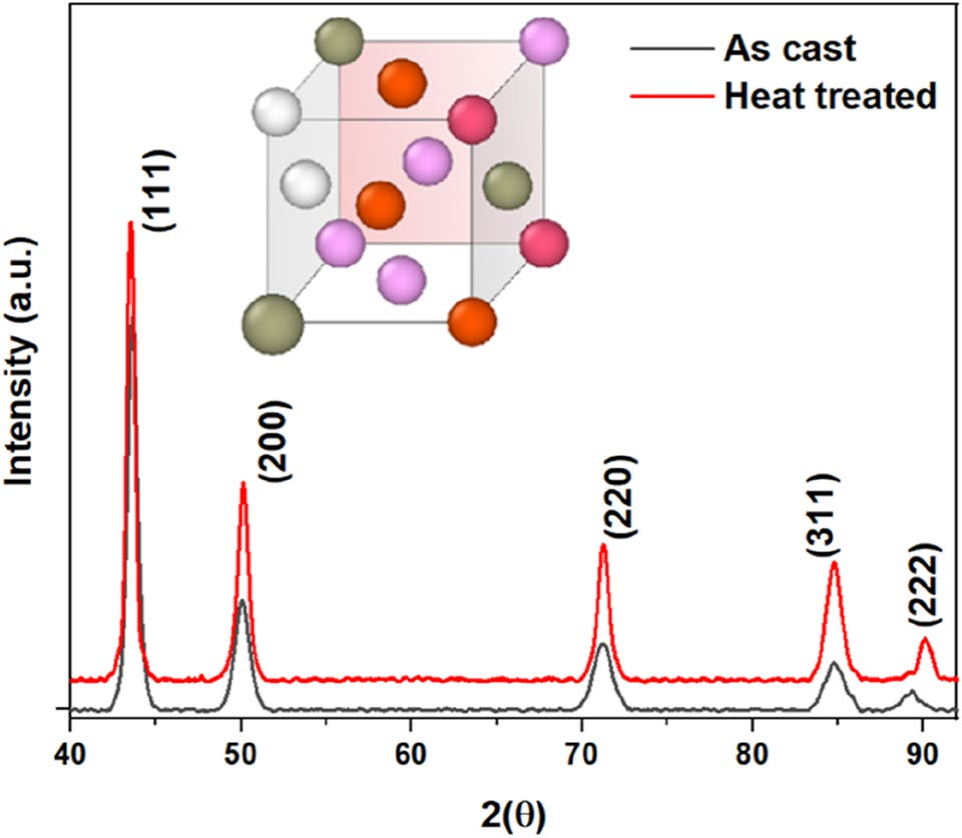
XRD analysis of newly synthesized HEA (Ni_25_Cu_18.75_Fe_25_Co_25_Al_6.25_) for as-cast and heat-treated sample. Peaks (111), (200), (220), (311) and (222) correspond to the FCC structure.

X-ray diffraction (XRD, Broker D8) was used to identify the phase of Ni_25_Cu_18.75_Fe_25_Co_25_Al_6.25_ alloy_,_ with the wavelength Cu Kα (λ = 1.54056 Å) at a step size of 0.02° recorded with angles (*2θ)* in the range of 20°–100° (see Fig. 8). The Bragg’s peaks (111), (200), (220), (311), and (222), belong to the lattice planes of FCC phase, while no other peaks corresponding to ordered structure were detected, indicating that this new HEA resides in a crystalline FCC structure.

**Figure 9:**
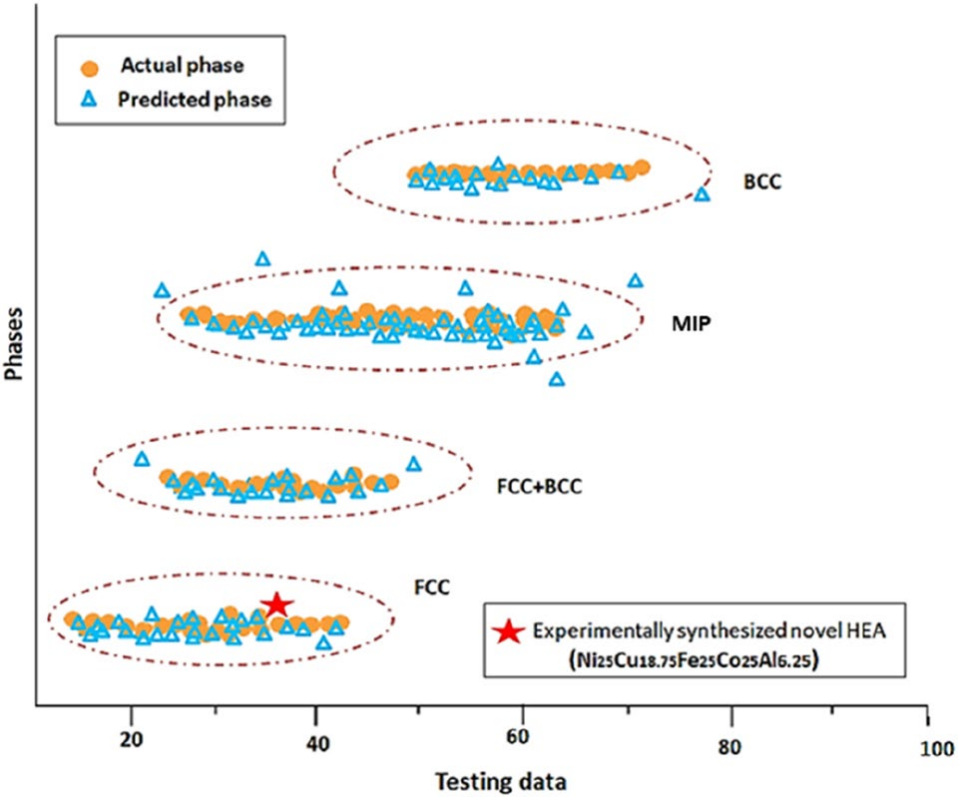
Phase prediction of novel HEA composition Ni_25_Cu_18.75_Fe_25_Co_25_Al_6.25_ (shown in red asterisk), along with 240 test data.

Figure 9 compares various HEAs for the test datasets (240 alloys) from original data with the newly developed and synthesised HEA composition Ni_25_Cu_18.75_Fe_25_Co_25_Al_6.25_. The orange dot represents the reported experimental phase of the HEA for the test data while the blue triangles represent the RFC prediction, and the red asterisk represents the new composition of the Ni_25_Cu_18.75_Fe_25_Co_25_Al_6.25_. RFC algorithm indicated that this new HEA would stabilise as FCC phase at room temperature.

A remarkable agreement between RFC model prediction and experiment can be seen for this new composition of HEA. It can be inferred that the V-RFC model is reliable and robust in predicting phases of novel compositions of HEAs as simple solid solution (FCC, BCC, FCC + BCC) and MIP (Mixture of intermetallic phases) with higher reliability of phase prediction, where MIP denotes the presence of either pure IM compounds such as α, β, σ, L12, L21, C15, C15, C36 Laves or mixture of IM + SS phases (FCC + IM, FCC + BCC + α, BCC + IM, FCC + α + β, BCC + Laves, BCC1 + BCC2 + C15 Laves, BCC + β-ordered BCC, FCC + CoMo2Ni-type IM, FCC + IM etc.). However, this method is limited in exactly interpreting the number and types of phases present in a complex multiphase HEA, which it usually predicts as MIP phase, but it is robust for predicting solid-solution phases.

A follow-on work of this study will be to test and predicting the mechanical properties of HEAs which will require atomistic studies on HEA. On surveying the wealth of literature in the arena of molecular dynamics simulation, the EAM potential currently available for 16 elements namely, Cu, Ag, Au, Ni, Pd, Pt, Al, Pb, Fe, Mo, Ta, W, Mg, Co, Ti, Zr was tested by the authors and found robust to predict HEAs mechanical properties reliably^80^. In keeping this momentum for the purpose of traceability, we have taken the same alloy used in this MD study for the purpose of ML validation in this work as well (Ni_25_Cu_18.75_Fe_25_Co_25_Al_6.25_).

## Conclusion

This study attempts to develop a novel high entropy alloy through comparison of prior literature using robust machine learning algorithms. An effort in this area will strengthen the materials discovery research to guide the initiatives at the forefront of Materials-4.0. Major conclusions from this study can be summarised as:

- An imbalanced dataset involving synthetic data merged into the experimental data can lead to spurious outcomes when feeding to the machine learning algorithms. An attempt like this (which has been routinely done in literature) can although help achieve higher accuracy from the model, but it can compromise the quality of prediction, particularly, while inferring complex phases of high entropy alloys (HEAs). Our novel work using machine learning revealed that it is possible to make reliable predictions to infer phase information of an HEA merely by using five crucial parameters (Valence electron concentration (VEC), Electronegativity difference (∆χ), Mixing entropy (∆S_mix_), Atomic size difference (δ), and Mixing enthalpy (∆H_mix_)). One must however be cautious of using selectively screened input experimental data to feed the ML algorithm.
- The performance of ML models was assessed using accuracy, precision, recall, f1-score, ROC-AUC score and tenfold cross-validation scores. Across, K-nearest neighbours (V-KNN), support vector machine (V-SVM), decision tree classifier (V-DTC), random forest classifier (V-RFC) and XGBoost (V-XGB), Random Forest Classifier (V-RFC) model performed the best in correctly predicting the phase of an alloy as solid-solutions (FCC, BCC, FCC + BCC) or MIP which denotes the presence of either pure IM compounds (such as α, β, σ, L12, L21, C15, C15, C36 Laves) or mixture of IM + SS phases (such as FCC + IM, FCC + BCC + α, BCC + IM, FCC + α + β, BCC + Laves, BCC1 + BCC2 + C15 Laves, BCC + β-ordered BCC, FCC + CoMo2Ni-type IM, FCC + IM etc.), with an average accuracy of 84%, ROC-AUC score of 0.9649, tenfold cross-validation mean score of 0.9315. Thus, V-RFC model can be used for predicting phases of new HEAs as solid-solution (FCC, BCC, FCC + BCC) or MIP (Mixture of Intermetallics phases). This claim was reinforced by comparing the V-RFC predicted phases with experimental phases reported recently for the newly developed HEAs, where V-RFC correctly predicted solid solution phases (BCC and FCC) for 2 refractory HEAs^10^, and 2 precious metal HEAs^63^ respectively. The phase of 3d-transition metal HEA (Al_0.5_CrCuNiV)^62^ was also correctly predicted as MIP, as per the considered assumption, however the actual phase contained 1FCC + 2BCC + ordered B2. Note that our algorithm worked robustly in predicting solid-solution phases, and complex multiphase HEAs as MIP, but it was found limited in interpreting the number and types of phases present in a complex multiphase HEA.
- Although there are few studies reporting higher accuracy from the models using synthetic data, we showed that this can lead to inaccurate predictions. For instance, care must be taken while extracting the data from mixed manufacturing routes and tackling an imbalanced dataset. This becomes clear from the fact that although the ANN model used in Bakr et al.^28^ study achieved an accuracy of 93.4% but could not correctly predicted the existence of amorphous phase. Hence, proving the fact that even after achieving 93.4% of accuracy, their model resulted in erroneous predictions while treating the imbalanced data. Also. the recall, precision, and F1-score for amorphous (AM) phase were not defined clearly. It was also acknowledged by Risal et al*.*^51^ that the “ML algorithms usually do not perform well for imbalanced dataset” and reported 92.31% accuracy by using oversampling method to balance out the minority class data by polluting it with synthetic data. Accordingly, we explored the use of SMOTE-Tomek link to resample our dataset in support of testing our claim, using RFC model (ST-RFC). An average accuracy of 92% was observed on augmented data of 1392 instances (1200 + 192) for ST-RFC model. Although, a great increment in accuracy was observed, but it could not yield better phase predictions.
- Using the robust RFC algorithm developed in this work, we report the development of a novel HEA with its composition Ni_25_Cu_18.75_Fe_25_Co_25_Al_6.25_. The peaks from X-ray diffraction revealed an FCC structure in corroboration with the ML predictions.

## Supplementary Information

Supplementary Information.

## Data Availability

The data is available from https://gitfront.io/r/user-6296136/ErgmsuZSHXiG/Phase-prediction-of-HEAs-private-share/blob/HEA%20dataset-1200%20instances-NEW.xlsx. The source of data was from the literature^30–32^.

## Abbreviations

*AM*: Amorphous phase
*AUC *: Area under the ROC curve
*BCC*: Body centered cubic
*c*_*i*_: Concentration of the *i*th element
*DTC*: Decision tree classifier
*FCC*: Face centered cubic
*FCC* + *BCC*: Mixed FCC and BCC solid solutions
*FP*: False positives
*FN*: False negatives
FPR: False positive rate
*HCP*: Hexagonal close packed
*IM*: Intermetallic phase
*KNN *: K-nearest neighbours
*n*: Total number of metallic elements in a high entropy alloy
*RFC*: Random forest classifier
*ROC *: Receiver operating characteristic
*r*_*i*_: Atomic radius of the *i*th element,
*r̄*: Average atomic radius
*SVM*: Support vector machine
*SS*: Solid-solution
*MIP*: Mixture of intermetallic phases.
*TP*: True positives
*TN *: True negatives
*TPR*: True positive rate
*VEC*: Valence electron concentration
*x*_*i*_: Pauling electronegativity
*x̄*: Averaged Pauling electronegativity
XGB: XGBoost/extreme gradient boosting
*∆**χ*: Electronegativity difference
*δ*: Atomic size difference
*∆**H*_*mix*_: Mixing enthalpy
*∆**S*_*mix*_: Mixing entropy

## Code availability

The code used for training and testing various machine learning algorithms: K-nearest neighbour, Support Vector Machine, Decision Tree Classifier, Random Forest Classifier, and XGBoost are available from https://gitfront.io/r/user-6296136/ErgmsuZSHXiG/Phase-prediction-of-HEAs-private-share/ .The code for model interrogation (that supports the findings of this study) can be made available upon reasonable request.

## References

[1] George EP, Raabe D, Ritchie RO (2019). High-entropy alloys. Nat. Rev. Mater..

[2] Ye Y (2016). High-entropy alloy: Challenges and prospects. Mater. Today.

[3] Pickering EJ, Jones NG (2016). High-entropy alloys: A critical assessment of their founding principles and future prospects. Int. Mater. Rev..

[4] Katiyar NK (2021). A perspective on the catalysis using the high entropy alloys. Nano Energy.

[5] Li Z (2016). Metastable high-entropy dual-phase alloys overcome the strength–ductility trade-off. Nature.

[6] Cantor B (2021). Multicomponent high-entropy Cantor alloys. Prog. Mater Sci..

[7] Cantor B (2014). Multicomponent and high entropy alloys. Entropy.

[8] Murty, B.S., et al., *High-entropy alloys* (Elsevier, 2019).

[9] Katiyar NK, Goel G, Goel S (2021). Emergence of machine learning in the development of high entropy alloy and their prospects in advanced engineering applications. Emerg. Mater..

[10] Han Z (2018). Microstructures and mechanical properties of TixNbMoTaW refractory high-entropy alloys. Mater. Sci. Eng., A.

[11] Pan Y (2021). New insights into the methods for predicting ground surface roughness in the age of digitalisation. Precis. Eng..

[12] Jose R, Ramakrishna S (2018). Materials 4.0: Materials big data enabled materials discovery. Appl. Mater. Today.

[13] Lederer Y (2018). The search for high entropy alloys: A high-throughput ab-initio approach. Acta Mater..

[14] Sun X (2017). Phase selection rule for Al-doped CrMnFeCoNi high-entropy alloys from first-principles. Acta Mater..

[15] Liu X (2021). Monte Carlo simulation of order-disorder transition in refractory high entropy alloys: A data-driven approach. Comput. Mater. Sci..

[16] Gao MC (2017). Computational modeling of high-entropy alloys: Structures, thermodynamics and elasticity. J. Mater. Res..

[17] Wu M (2020). CALPHAD aided eutectic high-entropy alloy design. Mater. Lett..

[18] Pyzer-Knapp EO (2022). Accelerating materials discovery using artificial intelligence, high performance computing and robotics. NPJ Comput. Mater..

[19] Schmidt J (2019). Recent advances and applications of machine learning in solid-state materials science. NPJ Comput. Mater..

[20] Ourmazd A (2020). Science in the age of machine learning. Nat. Rev. Phys..

[21] Kailkhura B (2019). Reliable and explainable machine-learning methods for accelerated material discovery. NPJ Comput. Mater..

[22] Cai J (2020). Machine learning-driven new material discovery. Nanoscale Adv..

[23] Jiang L (2016). Formation rules of single phase solid solution in high entropy alloys. Mater. Sci. Technol..

[24] Guo S (2015). Phase selection rules for cast high entropy alloys: an overview. Mater. Sci. Technol..

[25] Borg CKH (2020). Expanded dataset of mechanical properties and observed phases of multi-principal element alloys. Sci. Data.

[26] Gorsse S (2018). Database on the mechanical properties of high entropy alloys and complex concentrated alloys. Data Brief.

[27] Himanen L (2019). Data-driven materials science: Status. Challenges, Perspect..

[28] Bakr M, Syarif J, Hashem IAT (2022). Prediction of phase and hardness of HEAs based on constituent elements using machine learning models. Mater. Today Commun..

[29] Martin P (2022). HEAPS: A user-friendly tool for the design and exploration of high-entropy alloys based on semi-empirical parameters. Comput. Phys. Commun..

[30] Miracle DB, Senkov ON (2017). A critical review of high entropy alloys and related concepts. Acta Mater..

[31] Machaka R (2021). Machine learning-based prediction of phases in high-entropy alloys: A data article. Data Brief.

[32] Precker, C.E.G.C., Andrea, Landín, M., *Materials for design open repository. high entropy alloys *(2021).

[33] Zhang Y (2008). Solid-solution phase formation rules for multi-component alloys..

[34] Takeuchi A, Inoue A (2001). Quantitative evaluation of critical cooling rate for metallic glasses. Mater. Sci. Eng., A.

[35] Sheng G, Liu CT (2011). Phase stability in high entropy alloys: Formation of solid-solution phase or amorphous phase. Prog. Nat. Sci. Mater. Int..

[36] Katrutsa A, Strijov V (2017). Comprehensive study of feature selection methods to solve multicollinearity problem according to evaluation criteria. Expert Syst. Appl..

[37] Cuartas M (2021). Machine learning algorithms for the prediction of non-metallic inclusions in steel wires for tire reinforcement. J. Intell. Manuf..

[38] Fan C (2021). A review on data preprocessing techniques toward efficient and reliable knowledge discovery from building operational data. Front. Energy Res..

[39] Famili A (1997). Data preprocessing and intelligent data analysis. Intell. Data Anal..

[40] Cunningham P, Delany SJ (2021). k-Nearest Neighbour Classifiers—A Tutorial. ACM Comput. Surv..

[41] Cover T, Hart P (1967). Nearest neighbor pattern classification. IEEE Trans. Inf. Theory.

[42] Salam Patrous, Z. *Evaluating XGBoost for User Classification by using Behavioral Features Extracted from Smartphone Sensors*, in *TRITA-EECS-EX* (2018).

[43] Jakkula, V. *Tutorial on support vector machine (svm).* School of EECS, Washington State University. **37**(2.5), 3 (2006).

[44] Song YY, Lu Y (2015). Decision tree methods: Applications for classification and prediction. Shanghai Arch Psychiatry.

[45] Izza, Y., Ignatiev, A., Marques-Silva, J. On explaining decision trees. arXiv preprint arXiv:2010.11034 (2020).

[46] Breiman L (2001). Random forests. Mach. Learn..

[47] Biau G (2012). Analysis of a random forests model. J. Mach. Learn. Res..

[48] Chen, T., *et al.* Xgboost: extreme gradient boosting. *R package version* 0.4–2. **1**(4), 1–4 (2015).

[49] Akosa, J. *Predictive accuracy: A misleading performance measure for highly imbalanced data*. in *Proceedings of the SAS global forum* (2017).

[50] Luque A (2019). The impact of class imbalance in classification performance metrics based on the binary confusion matrix. Pattern Recogn..

[51] Gu, Q., Zhu, L., & Cai, Z. *Evaluation measures of the classification performance of imbalanced data sets*. In *International symposium on intelligence computation and applications* (Springer, 2009).

[52] Kulkarni A, Chong D, Batarseh FA (2020). Foundations of data imbalance and solutions for a data democracy. data democracy.

[53] Thölke, P., *et al.* Class imbalance should not throw you off balance: Choosing classifiers and performance metrics for brain decoding with imbalanced data. bioRxiv (2022). 10.1016/j.neuroimage.2023.120253 37385392

[54] Pedregosa F (2011). Scikit-learn: Machine learning in Python. J. Mach. Learn. Res..

[55] Hossin M, Sulaiman MN (2015). A review on evaluation metrics for data classification evaluations. Int. J. Data Min. Knowl. Manag. Process.

[56] Bewick V, Cheek L, Ball J (2004). Statistics review 13: Receiver operating characteristic curves. Crit. Care.

[57] Varpa K (2011). Applying one-vs-one and one-vs-all classifiers in k-nearest neighbour method and support vector machines to an otoneurological multi-class problem. User Centred Networked Health Care.

[58] Yacouby, R., & Axman, D. *Probabilistic extension of precision, recall, and F1 score for more thorough evaluation of classification models*. In *Proceedings of the first workshop on evaluation and comparison of NLP systems* (2020).

[59] Goutte, C., & Gaussier, E. *A probabilistic interpretation of precision, recall and F-score, with implication for evaluation*. in *European conference on information retrieval* (Springer, 2005).

[60] Risal S (2021). Improving phase prediction accuracy for high entropy alloys with machine learning. Comput. Mater. Sci..

[61] Batista, G.E., Bazzan, A.L., & Monard, M.C. *Balancing Training Data for Automated Annotation of Keywords: a Case Study*. in *WOB* (2003).

[62] Yi J (2020). A novel Al0 5CrCuNiV 3d transition metal high-entropy alloy: Phase analysis, microstructure and compressive properties. J. Alloys Compounds.

[63] Sohn S (2017). Noble metal high entropy alloys. Script. Mater..

[64] Mandal P (2022). Phase prediction in high entropy alloys by various machine learning modules using thermodynamic and configurational parameters. Metals Mater. Int..

[65] Zhu W (2022). Phase formation prediction of high-entropy alloys: a deep learning study. J. Market. Res..

[66] Jaiswal UK (2021). Machine learning-enabled identification of new medium to high entropy alloys with solid solution phases. Comput. Mater. Sci..

[67] Krishna YV, Jaiswal UK, Rahul M (2021). Machine learning approach to predict new multiphase high entropy alloys. Script. Mater..

[68] Pei Z (2020). Machine-learning informed prediction of high-entropy solid solution formation: Beyond the Hume-Rothery rules. NPJ Comput. Mater..

[69] Lee SY (2021). Deep learning-based phase prediction of high-entropy alloys: Optimization, generation, and explanation. Mater. Des..

[70] Al-Shibaany, Z.Y.A., *et al.**Deep learning-based phase prediction of high-entropy alloys*. In *IOP Conference Series: Materials Science and Engineering*. 2020. IOP Publishing.

[71] Chau NH (2021). Phase prediction of multi-principal element alloys using support vector machine and bayesian optimization.

[72] Zhang Y (2020). Phase prediction in high entropy alloys with a rational selection of materials descriptors and machine learning models. Acta Mater..

[73] Zhou Z (2019). Machine learning guided appraisal and exploration of phase design for high entropy alloys. NPJ Comput. Mater..

[74] Agarwal, A., & Prasada Rao, A. Artificial intelligence predicts body-centered-cubic and face-centered-cubic phases in high-entropy alloys. *Jom*. **71**(10), 3424–3432 (2019).

[75] Li Y, Guo W (2019). Machine-learning model for predicting phase formations of high-entropy alloys. Phys. Rev. Mater..

[76] Choudhury, A., *et al.* Structure prediction of multi-principal element alloys using ensemble learning. *Eng. Comput.* (2019).

[77] Huang W, Martin P, Zhuang HL (2019). Machine-learning phase prediction of high-entropy alloys. Acta Mater..

[78] Islam N, Huang W, Zhuang HL (2018). Machine learning for phase selection in multi-principal element alloys. Comput. Mater. Sci..

[79] Tancret F (2017). Designing high entropy alloys employing thermodynamics and Gaussian process statistical analysis. Mater. Des..

[80] Fan P (2022). Uniaxial pulling and nano-scratching of a newly synthesized high entropy alloy..

