# Supplementary material for: Phase prediction and experimental realisation of a new high entropy alloy using machine learning
Source: Sci Rep. 2023 Mar 23;13:4811. doi: 10.1038/s41598-023-31461-7 (PMC10036487; doi:10.1038/s41598-023-31461-7)
Supplement: Supplementary file 1 — Supplementary Information. [file 41598_2023_31461_MOESM1_ESM.docx]

**Supplementary Information**

**Phase prediction and experimental realisation of a new high entropy alloy using machine learning**

**Swati Singh^a^, Nirmal Kumar Katiyar^b^, Saurav Goel^a,b,c+^ and Shrikrishna N. Joshi^a*^**

*^a^ Department of Mechanical Engineering, Indian Institute of Technology Guwahati, Guwahati, 781039, India*

*^b^ School of Engineering, London South Bank University, 103 Borough Road, London, SE1 0AA, UK*

*^c^University of Petroleum and Energy Studies, Dehradun, 248007, India*

*Corresponding author(s): ^+^GoeLs@Lsbu.ac.uk and* [**snj@iitg.ac.in*](mailto:*snj@iitg.ac.in)

A representative dataset of an imbalanced nature containing a total of 1200 entries showing 441 compositions of ‘MIP’ i.e., mixture of intermetallic phases, 372 compositions of BCC solid-solution ‘BCC_SS’ phase, 220 compositions of FCC solid-solution ‘FCC_SS’ phase, and 167 compositions of mixed ‘FCC+BCC’ phase, all synthesized by melting and casting routes, were collected from their respective experimental study and literature [1-3]. The dataset contains a total of 35 different input feature variables (including the chemical composition and five fundamental physical parameters) and 1 target variable i.e., the resulting phase for each composition of HEAs, as depicted in Table S1.

**Table S1:** Representative dataset of HEA containing chemical composition and five most important physical features for phase prediction of HEAs used in this study.


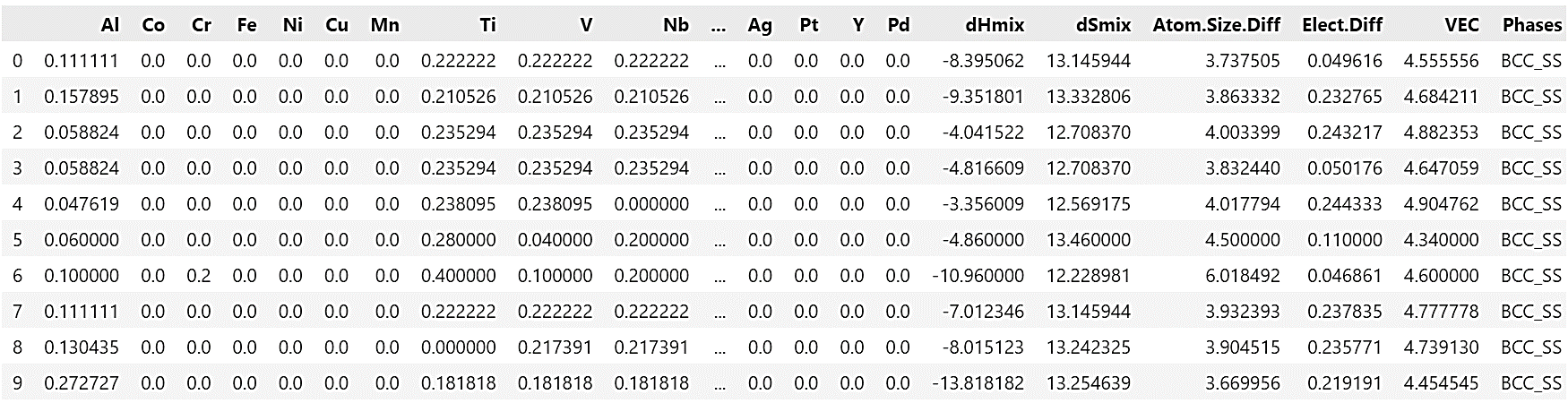

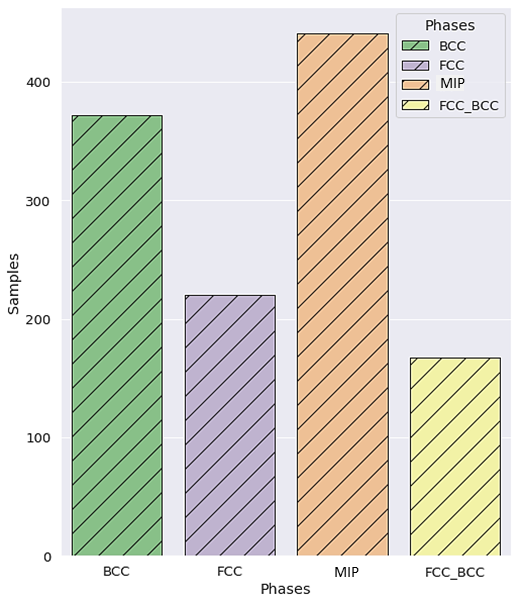

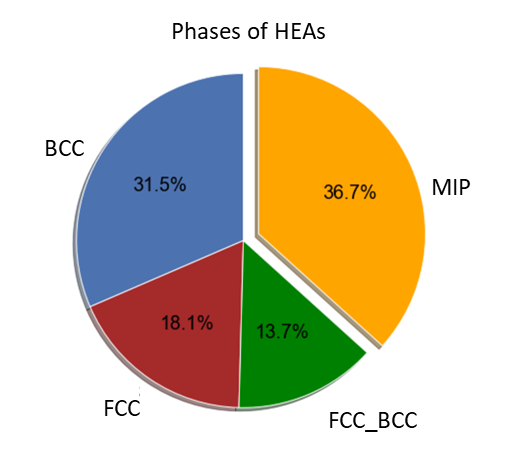


**Figure S1**: HEA dataset showing the (a) numbers, and (b) percentages of different phases of HEAs such as MIP, BCC_SS, FCC_SS, and FCC+BCC in the dataset.


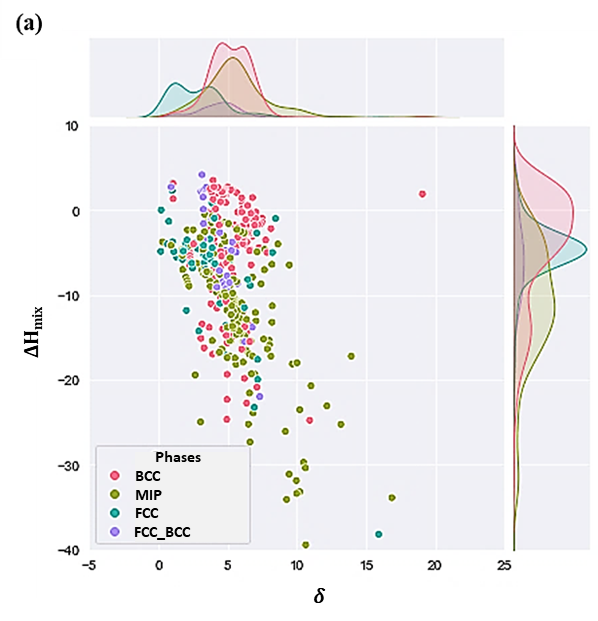


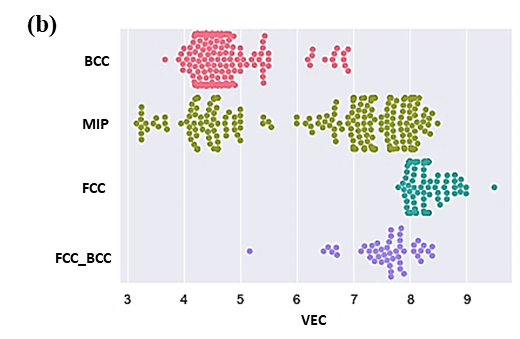


**Figure S2**: (a) Joint plot of dH_mix_ and (𝛿) for the dataset (b) swarm plot showing the role of VEC in BCC, FCC, MIP, and FCC+BCC phase selection, both fulfilling the HEAs criteria given by some empirical rules.

The current dataset satisfying these phase-selection rules is shown by **Figure S2**, with the joint plot of (∆H_mix_ –𝛿) and a swarm plot of VEC values for four different phases of HEAs.

**Table S2:** Characteristics and parameters of five ML models employed in this study.

| **ML Algorithm** | **Type** | **Description** |
| --- | --- | --- |
| K-nearest neighbor (KNN) | 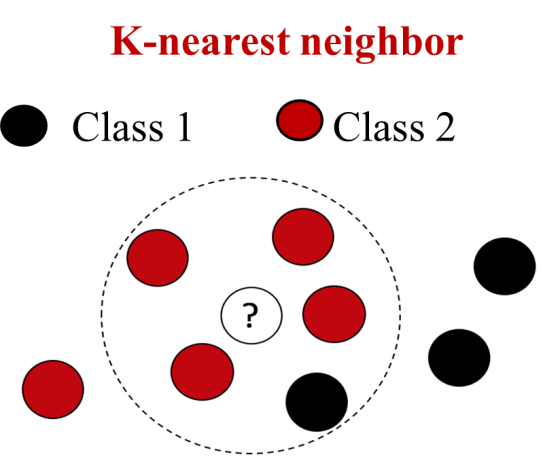 | It makes predictions by searching similar instances, as per the given value of k (according to Euclidean distance). Parameters K or n_neighbors=5, and metric= ‘euclidean’ are some of the important hyper-parameters of KNN algorithm set by default for this study. |
| Support Vector Machine (SVM) | 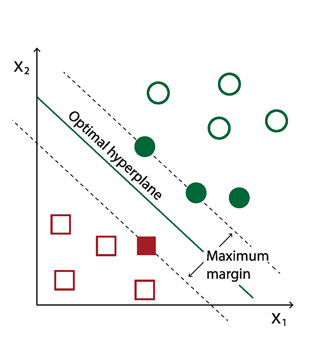 | In SVM model, a hyperplane is selected to separate two different classes, with the highest margin that best separates the two classes. The closest data points which define the margins are called **support vectors.** Hyper-parameters such as C (Regularization parameter) =1.0, and kernel= ‘rbf’ (radial basis function) are some of the important hyper-parameters, set by default. |
| Decision Tree Classifier (DTC) | Single Decision Tree  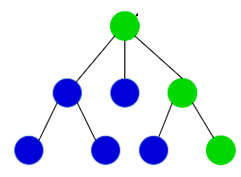 | Decision tree algorithm is based on the branching method to match all the possible outcomes based on the consequences of our decisions. Criterion= ‘gini’ for Gini impurity, max_depth=None, and min_sample_split=2, are some of the important hyper-parameters of DTC, set by default. |
| Random Forest Classifier (RFC) | 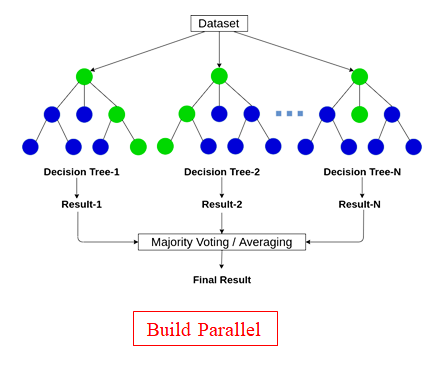 | Random forest algorithm is an ensemble of various decision trees, builds on different samples, and takes majority voting for classification. It uses Bootstrap Aggregating or Bagging method. Value of some important hyper-parameters such as, n_estimators=100, criterion=‘gini’, max_depth=None, and min_sample_split=2, are set by default. |
| XGBoost classifier (XGB) | 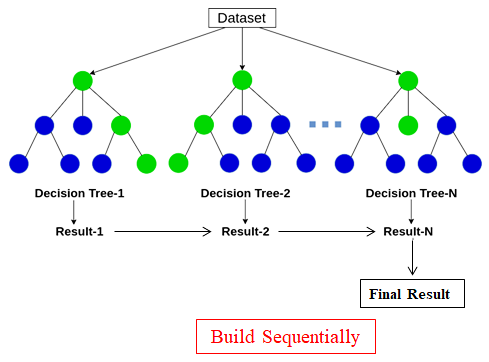 | XGBoost is an ensemble learning algorithm that provides a parallel tree boosting “boosting” or improving a single weak model by combining it with several other weak models in order to generate a collectively strong model.  learning_rate=0.30, max_depth=6, and n_estimators=100 are default hyper-parameters used in this study. |

**Table S3:** Evaluation metrics and their statistical definition used for an imbalanced multiclass classification problem.

| **Evaluation Metric Terms** | **Description** |
| --- | --- |
| True Positive (TP) | TP is the total number of right predictions when the actual class was positive. |
| True Negative (TN) | TN is the total number of right predictions when the actual class was negative. |
| False Positive (FP) | FP is the total number of wrong predictions when the actual class was positive. |
| False Negative (FN) | FN is the total number of wrong predictions when the actual class was negative. |
| Accuracy | $\frac{TP+TN}{TP+TN+FP+FN}$ |
| Precision | Precision tells how many of the correctly predicted classes actually turned out to be positive. |
| Recall | Recall, on the other hand, make sure that we are not missing out on the positive observations. |
| F1-score | F1-score is the harmonic mean of precision and recall, gives a combined idea about both metrics, calculated as follows:  F1-Score = $\frac{2 \times(Recall \times Precision)}{(Recall + Precision)}$ |
| True Positive Rate (TPR) | TPR= $\frac{\mathrm{TP}}{TP+FN}$ |
| False Positive Rate (FPR) | FPR= $\frac{\mathrm{TN}}{TN+FP}$ |

**Table S4:** Performance of vanilla models using various evaluation metrics.

| Algorithms | | Simple Imputer with different strategy | | | MICE Imputer | KNN Imputer |
| --- | --- | --- | --- | --- | --- | --- |
|  | Metrics | Mean | Median | Constant |  |  |
| Vanilla KNN  (V-KNN) | Accuracy | 68.75 | 68.75 | 68.75 | 68.75 | 68.75 |
|  | F1-score, Precision, Recall | 0.6876  0.6894  0.6875 | 0.6876  0.6894  0.6875 | 0.6876  0.6894  0.6875 | 0.6876  0.6894  0.6875 | 0.6876  0.6894  0.6875 |
|  | ROC-AUC/  10-fold CV score | 0.9453, 0.8341 | 0.9453, 0.8341 | 0.9453, 0.8341 | 0.9453, 0.8341 | 0.9453, 0.8341 |
| Vanilla SVM  (V-SVM) | Accuracy | 71.25 | 71.25 | 71.25 | 71.25 | 71.25 |
|  | F1-score,  Precision, Recall | 0.7113  0.7189  0.7125 | 0.7113  0.7189  0.7125 | 0.7113  0.7189  0.7125 | 0.7113  0.7189  0.7125 | 0.7113  0.7189  0.7125 |
|  | ROC-AUC/  10-fold CV score | 0.9117, 0.8762 | 0.9055, 0.8750 | 0.9053, 0.8750 | 0.8846, 0.8747 | 0.9052, 0.8739 |
| Vanilla DTC  (V-DTC) | Accuracy | 79.17 | 79.58 | 77.50, | 79.58 | 8042 |
|  | F1-score,  Precision, Recall | 0.7959  0.7960  0.7958 | 0.7958  0.7959  0.7958 | 0.7750  0.7753  0.7750 | 0.7916  0.7916  0.7917 | 0.8043  0.8049  0.8042 |
|  | ROC-AUC/  10-fold CV score | 0.7947, 0.7818 | 0.8129, 0.7772 | 0.8173, 0.7841 | 0.836, 0.7828 | 0.8275, 0.7819 |
| Vanilla RFC  (V-RFC) | Accuracy | 82.834 | 82.5 | 82.25 | 82.91 | 84.5 |
|  | F1-score,  Precision, Recall | 0.8214  0.8205  0.8225 | 0.8215  0.8132  0.8203 | 0.8203  0.8231  0.8250 | 0.8359  0.8347  0.8450 | 0.8172  0.8264  0.8167 |
|  | ROC-AUC/  10-fold CV score | 0.9566, 0.9251 | 0.9546, 0.9238 | 0.9550, 0.9242 | 0.9349, 0.9315 | 0.9522, 0.9272 |
| Vanilla XGB  (V-XGB) | Accuracy | 0.8250 | 0.8250 | 0.8250 | 0.8250 | 0.8250 |
|  | F1-score, Precision, Recall | 0.8215  0.8232  0.8250 | 0.8215  0.8232  0.8250 | 0.8215  0.8232  0.8250 | 0.8215  0.8232  0.8250 | 0.8215  0.8232  0.8250 |
|  | ROC-AUC/  10-fold CV score | 0.9455, 0.8998 | 0.9455, 0.8998 | 0.9455, 0.8998 | 0.9453, 0.9074 | 0.9455, 0.8998 |

**Table S5:** High-entropy alloys (that were not the part of train-test set) correctly predicted as MIP using RFC model as per considered assumption, but the number and types of phases could not be interpreted.

| **Alloy** | **V-RFC predicted phases** | **Actual phase** | **References** |
| --- | --- | --- | --- |
| CoCrCuFeMnNiTiV | MIP | FCC+BCC+IM | [4] |
| Al0.2Co1.5CrFeNi1.5Ti | MIP | Gamma (FCC) + η (Ni, Co)_3_Ti | [5] |
| Ti_20_Zr_20_Hf_20_Nb_20_Cr_20_ | MIP | BCC + 2 Laves (Cr_2_Nb and Cr_2_Hf) | [6] |
| Ni_44.8_(FeCoCr)_40_(AlTi)_15_Hf_0.2_ | MIP | FCC+ordered fcc phase (L1_2_) | [7] |
| AlTiCrFeNiCu | MIP | BCC1+BCC2+Fe2Ti | [8] |
| AlCoCrFeNiTi0.4 | MIP | A2 (disordered BCC)+B2 (ordered BCC)+Laves | [9] |
| AlCoCrFeNb0.25Ni | MIP | BCC + (Laves + bcc) | [10] |

**References**

1. Machaka, R., et al., *Machine learning-based prediction of phases in high-entropy alloys: A data article.* Data in brief, 2021. **38**.

2. Precker, C.E.G.C., Andrea; Muíños Landín, Santiago, *Materials for Design Open Repository. High Entropy Alloys.* 2021.

3. Miracle, D.B. and O.N. Senkov, *A critical review of high entropy alloys and related concepts.* Acta Materialia, 2017. **122**: p. 448-511.

4. Zhou, Y., et al., *Microstructure and compressive properties of multicomponent Alx (TiVCrMnFeCoNiCu) 100− x high-entropy alloys.* Materials Science and Engineering: A, 2007. **454**: p. 260-265.

5. Chuang, M.-H., et al., *Microstructure and wear behavior of AlxCo1. 5CrFeNi1. 5Tiy high-entropy alloys.* Acta Materialia, 2011. **59**(16): p. 6308-6317.

6. Fazakas, E., et al., *Experimental and theoretical study of Ti20Zr20Hf20Nb20X20 (X= V or Cr) refractory high-entropy alloys.* International Journal of Refractory Metals and Hard Materials, 2014. **47**: p. 131-138.

7. Zhang, L., et al., *The microstructure and high-temperature properties of novel nano precipitation-hardened face centered cubic high-entropy superalloys.* Scr. Mater, 2018. **146**: p. 226-230.

8. Pi, J.-H., et al., *Microstructure and property of AlTiCrFeNiCu high-entropy alloy.* Journal of Alloys and Compounds, 2011. **509**(18): p. 5641-5645.

9. Jiao, Z., et al., *Superior mechanical properties of AlCoCrFeNiTi x high-entropy alloys upon dynamic loading.* Journal of Materials Engineering and Performance, 2016. **25**(2): p. 451-456.

10. Ma, S. and Y. Zhang, *Effect of Nb addition on the microstructure and properties of AlCoCrFeNi high-entropy alloy.* Materials Science and Engineering: A, 2012. **532**: p. 480-486.
